# Functional circuits of LYL1 controlled by supraphysiological androgen in prostate cancer cells to regulate cell senescence

**DOI:** 10.1186/s12964-024-01970-7

**Published:** 2024-12-12

**Authors:** Mehdi Heidari Horestani, Katrin Schindler, Aria Baniahmad

**Affiliations:** Institute of Human Genetics, Jena University Hospital, Am Klinikum 1, 07740 Jena, Germany

**Keywords:** Androgen receptor, Androgen-induced cellular senescence, Prostate cancer, LYL1

## Abstract

**Background:**

Prostate cancer (PCa) is a public health problem mostly reported in developed countries. The androgen receptor (AR) regulates the development and physiological function of normal prostate as well as the proliferation of cancerous prostate tissue. Treatment with supraphysiological androgen levels (SAL) is used in bipolar androgen therapy and inhibits PCa growth, suggesting SAL induces a tumor suppressive program. It was shown that SAL induces cellular senescence, in PCa cell lines, human tumor samples and in xenografted mouse tumor model.

**Methods:**

Transcriptome and ChIP-seq analysis, PCa spheroids, knockdown (KD), co-immunoprecipitation, qRT-PCR, immune detection, in situ histochemistry.

**Results:**

Here we show that LYL1 is upregulated by the clock gene BHLHE40 in both C4-2 and LNCaP cells and mediates SAL-induced cellular senescence. LYL1 is a transcriptional co-factor with oncogenic activity in leukemia. However, analysis of a large cohort of PCa patients shows that LYL1 expression is reduced during PCa development and reduced expression is significantly associated with reduced overall survival. SAL induces the expression of LYL1 through upregulation of BHLHE40. On the other hand, the KD of *LYL1* enhances BHLHE40 expression via a negative feedback loop including p27kip1. Regulatory feedback loops were identified by rescue experiments. Functional analysis revealed that KD of *BHLHE40* reduces whereas *LYL1* KD enhances p27kip1 levels. The KD of p27kip1 suggests that this cell cycle inhibitor is a mediator of cellular senescence by the BHLHE40 - LYL1 regulatory loop. Interestingly, ChIP-seq data revealed recruitment of both AR and BHLHE40 to the LYL1 gene indicating that LYL1 is a novel direct target of both factors. Furthermore, RNA-seq data from C4-2 cells suggests that LYL1 and BHLHE40 encompass a large overlap of genes by SAL suggesting a co-regulatory activity controlled by androgens. In line with this, co-immunoprecipitation suggests LYL1 is in a complex with BHLHE40 and the AR.

**Conclusions:**

Three novel feed-back loops and a novel AR- BHLHE40 / LYL1 -p27kip1 axis has been identified mediating cellular senescence in PCa cells.

**Graphical Abstract:**

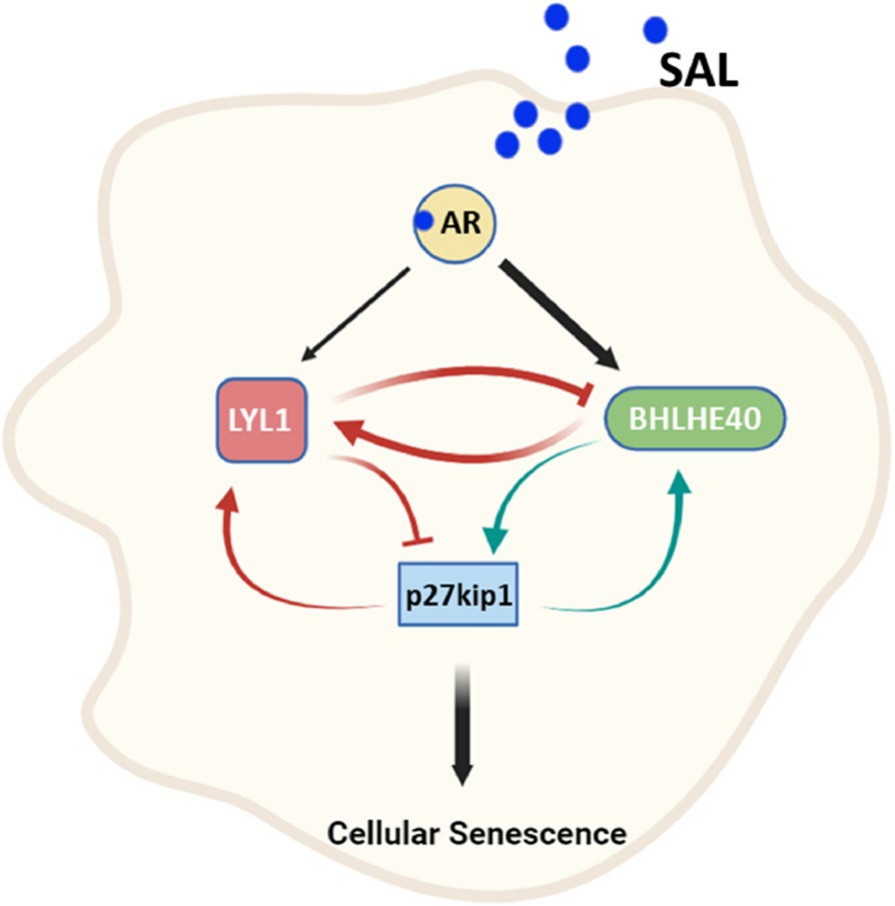

**Supplementary Information:**

The online version contains supplementary material available at 10.1186/s12964-024-01970-7.

## Background

PCa is a public health problem mostly reported in developed countries. It is the fifth most common cause of male tumors in the world and the second most common cause of cancer death in men [1]. The androgen receptor (AR) regulates the development and physiological function of normal prostate as well as the proliferation of the cancerous prostate tissue. Interestingly, the AR has both oncogenic and tumor suppressive functions [2]. PCa is initially an androgen-sensitively growing tumor that eventually becomes castration-resistant. While age is a major risk factor for this disease, the expression of AR is a key factor for tumor proliferation, not only in androgen-sensitive but also in castration resistant PCa cells. AR-targeted therapy using androgen deprivation therapy in combination with AR antagonists [3] is commonly used to block AR activity and slowing-down tumor growth. Thus, AR is an important drug target for AR-positive cancers [4, 5]. Paradoxically, treatment with supraphysiological androgen levels (SAL) also inhibits PCa growth, especially for PCa cells that grow in low androgen levels [6]. This observation has led to clinical trials termed bipolar androgen therapy (BAT), which uses cycles of SAL in combination with androgen deprivation currently being in phase II trials such as TRANSFORMER and RESTORE for patients with castration resistant PCa (CRPC) [7–9]. SAL has been shown to effectively inhibit cancer cell growth in cell lines, ex vivo treated patient prostatectomy specimen and PCa xenografts [6, 10, 11]. This suggests that SAL induces a tumor suppressive program in PCa cells [12]. Both the natural and synthetic androgens induce cellular senescence in a concentration-dependent manner at supraphysiological level [6, 11]. 10nM DHT, as a natural ligand, or 1nM R1881, as a synthetic ligand for AR, strongly inhibit growth [13] were defined as SAL and induce cellular senescence in PCa cell lines and induce cellular senescence in ex vivo treated patient sample [14–16]. We have previously shown some molecular pathways mediated by SAL to induce cellular senescence in adherent 2D-cultured human PCa cell lines and 3D spheroids [6, 17–20].

Here, we analyzed SAL-mediated pathways to induce cellular senescence in both castration sensitive PCa (CSPC) and CRPC cell lines. LYL1 with UniProt ID “P12980”, also known as bHLHa18, is a member of basic helix loop helix proteins [21]. There are two classes of bHLH proteins. Class I includes proteins that homo- and heterodimerize while class II only generate heterodimers with class I proteins like LYL1 [22]. It was shown that LYL1 preferentially co-activates gene expression in acute myeloid leukemia (AML) [23]. While most published studies address LYL1 in blood cancers or bone marrow, the role of LYL1 in other types of cancer such as PCa is not well understood.

Our group previously showed that the clock gene BHLHE40, which is a transcription factor and a member of bHLH proteins, induces cellular senescence in PCa cells [20]. Our RNA-seq data from KD of *BHLHE40* in PCa cells suggest the regulation of LYL1 by BHLHE40. Therefore, we hypothesized that LYL1 regulates SAL-mediated cellular senescence in PCa cells. Bioinformatic analyses indicate a functional relationship of LYL1 and BHLHE40 in SAL-induced cellular senescence that we confirmed by KD experiments. The obtained data suggest a novel regulatory pathway that includes the axis of AR-LYL1 / BHLHE40-p27kip1 to induce cellular senescence in both CSPC and CRPC. In addition, we identified three feedback loops in the SAL-mediated senescence inducing program.

## Methods

### Cell culture and treatments

C4-2 cell line representing castration resistant prostate cancer and LNCaP as castration sensitive cells were cultured in DMEM and RPMI medium respectively. Mediums were supplemented with 5% FBS, 1% penicillin/ streptomycin, 1% sodium pyruvate and 2.5% 1 M HEPES (pH 7.5). In addition, 20% F12 Nutri-mix for DMEM medium was included. 1nM R1881 in complete DMEM medium containing 5% untreated FBS, representing SAL [6, 15] or 0.1% DMSO as a solvent control was used as treatment.

### siRNA transfection

ON-TARGETplus Human siRNA targeting *LYL1*, *CDKN1B* or BHLHE40 was used for KD of targets at mRNA level in both cell lines according to the manufacturer’s protocol (Dharmacon). Moreover, ON-TARGETplus nontargeting control siRNA was used as a control. siRNAs were transfected by the DharmaFECT reagent #3 (Dharmacon), siRNAs sequences are listed in Table 1. Briefly, cells were cultured in antibiotic free medium a day prior to transfection. 16 h after seeding, cells were transfected with siRNA for 24 h. Next step, depending on the experiment’s plan, treatments were performed for 48–72 h.

**Table 1:** siRNA sequence (5’ …. 3’)

LYL1	1) CGGUUGAAGCGGAGACCAA 2) GAACGAGGUGCUCCGCCUA 3) CCAUGAAGUACAUCGGCUU 4) UCAACAGUGUCUACAUUGG
*CDKN1B*	1) CAAACGUGCGAGUGUCUAA 2) GCAGCUUGCCCGAGUUCUA 3) ACGUAAACAGCUCGAAUUA 4) GCAAUGCGCAGGAAUAAGG
*BHLHE40*	1) AAAGAGACGUGACCGGAUU 2) GAGAAAGGAUCGGCGCAAU 3) CCGAACAUCUCAAACUUAC 4) CCCGGGAGGUGCUUCAGUA
Non-targeting *siRNA*	1) UGGUUUACAUGUCGACUAA 2) UGGUUUACAUGUUGUGUGA 3) UGGUUUACAUGUUUUCUGA 4) UGGUUUACAUGUUUUCCUA

### Senescence assays

For senescence assay, cells were seeded in 6 well plates and a day after transfection, treatment was performed with SAL or DMSO for 72 h. The staining was performed as described previously [6]. In short, cells were fixed with 1% glutaraldehyde for 5 min after removing medium and washing cells with 1X PBS. Staining solution was prepared as follows, 40mM citric acid/sodium phosphate buffer (pH 6.0), 1 mg/ml X-Gal (5-bromo-4-chloro-3-indolyl-β-D-galactopyranoside), 5mM potassium ferrocyanide, 5mM potassium ferricyanide, 150mM sodium chloride and 2mM magnesium chloride. Cells were kept in solution at 37 °C for 24 h or 48 h depending on C4-2 or LNCaP cell lines. Enzymatic activity of β-galactosidase was quantified by counting greenish blue cells using brightfield microscopy.

### Growth assays

Crystal violet staining was performed for indirect measurement of cell growth according to the previously described protocol [6]. Briefly, cells were fixed with 1% glutaraldehyde and stained with 0.1% crystal violet. Staining was washed from cells with 0.9%, w/v tri-sodium citrate, 2% HCl, 40% ethanol as Sörenson’s solution. Absorbance was measured with spectrophotometer at 590 nm.

### mRNA isolation and qRT-PCR

RNA extraction was performed as previously described [24]. Reverse transcriptase-qPCR was conducted by producing c-DNA with High-Capacity cDNA Reverse Transcription Kit and random primers (Applied Biosystems, USA). Then, the SsoFast EvaGreen Supermix (Bio-Rad, Germany), gene specific primers, and Bio-Rad CFX Duet Real Time PCR machine was used to measure the expression level of targeted genes. Primers are listed in Table 2.

**Table 2:** Target-specific primers for qRT-PCR (5’ …. 3’)

BHLHE40	FWD: ACTTACCTTGAAGCATGTGAAAGCA REV: CATGTCTGGAAACCTGAGCAGAA
*CDKN1B*	FWD: GGCCTCAGAAGACGTCAAAC REV: ACAGGATGTCCATTCCATGA
*LYL1*	FWD: CAACTCTCCACCCTGGGAACTG REV: TGGCCCAATGTAGACACTGTT
*E2F1*	FWD: GCAGAGCAGATGGTTATGG REV: GATCTGAAAGTTCTCCGAAGAG
*TBP*	FWD: GATCTTTGCAGTGACCCAGCATCA REV: CTCCAGCACACTCTTCTCAGC
*Tubulin*	FWD: TGGAACCCACAGTCATTGATGA REV: TGATCTCCTTGCCAATGGTGTA

### Protein isolation and Western blotting

Cells were collected in cold 1X PBS and centrifuged shortly at 2500 rpm. 80 µl NETN lysis buffer containing 20mM Tris-HCl (pH 8.0), 100mM NaCl, 1mM EDTA, 1% NP-40 and 1% Tergitol was used to lyses cells. Of note, 50mM NaF, 100 µM Na_3_VO_4_, and 10mM β-Glycerophosphate as phosphatase inhibitors were freshly added to the lysis buffer. Centrifugation at 12,000 g at 4 °C for 10 min was performed to obtain the cell extracts. Quantification of the protein concentration was measured with BCA assay by using Nanodrop ND-1000 Spectrophotometer. 30 µg protein extract was loaded into SDS-PAGE gels to separate proteins then all proteins were transferred to PVDF membranes. The membrane was blocked with skim milk for 1 h prior to incubation with primary antibodies. Anti- β-Actin antibody served as loading control. The detection was performed by ImageQuantTM LAS 4000 (GE Healthcare Bio-Sciences AB). All used antibodies are listed in Table 3.

**Table 3:** Primary antibodies

anti-LYL1	1:1000	Abcam
anti-BHLHE40	1:20000 for WB, 1:500 for Co-IP	NOVUS Biological
anti-AR	1:1000 for WB, 1:500 for Co-IP	Merck Millipore
anti-p27kip1	1:2000	Santa Cruz
anti-β-Actin	1:10000	Abcam
anti-c-PARP	1:1000	Cell Signaling
anti-Ki67	1:200	Biozol
anti-mouse IgG	1:10000	Cell Signaling
anti-rabbit IgG	1:10000	Cell Signaling
anti-rabbit IgG Alexa 546	1:1000	Thermo Fisher

### Tumor spheroid generation, SA β-Gal staining and immunofluorescence detection

3D spheroids of C4-2 cells with and without *LYL1* KD were generated according to the previous protocol [19, 25]. Briefly, cells were seeded in 6 well plates 24 h prior transfection. The day after transfection cells were re-seeded in 96-well ultralow attachment plates (PerkinElmer) and incubated at 37 °C, 5% CO_2_ to form a spheroid. Next day, treatment was performed with SAL or DMSO for 6 days and every 3 days treatment was refreshed. Spheroids were fixed with 4% PFA for 20 min at RT. After washing step, spheroids were incubated with SA β-Gal (senescence associated β-Galactosidase) staining solution overnight at 37 °C (with further detail above). Next day, spheroids were paraffin-embedded and sliced with microtome (2 μm). Then, slides were deparaffinized and dehydrated in Xylol for 10 min, 100% EtOH, 96% EtOH, 70% EtOH, each step two times and each time for 5 min. 1X citrate buffer was used in steamer to heat-induced antigen retrieval for 20 min. Then slides were permeabilized with 0.2% Triton X100 for 10 min. Using brightfield microscopy images were captured. For Ki67 staining, after permeabilization, spheroid cryo-microtome slices were incubated in normal goat serum (NGS) (Biozol; Germany) for 1 h at RT. Following the blocking step, anti-Ki67 antibody, as a proliferation marker, was loaded overnight. Secondary anti-rabbit IgG Alexa 546 antibody was used for 1 h at RT in dark. Nuclei staining was performed by using DAPI (Life Technologies; USA) solution (1 µg/ml in 1x PBS) for 10 min. Afterward, slices were covered with Fluoromount-G^®^ (SouthernBiotech; USA) and coverslips. Using laser scanning microscope (Carl Zeiss LSM 880) images were captured. Antibodies are listed in Table 3.

### Co-immunoprecipitation assays

Co-immunoprecipitation (Co-IP) experiments were conducted as previously described [26]. In summary, protein A magnetic beads were incubated with a specific antibody targeting BHLHE40, AR, or normal rabbit IgG as a negative control. After thoroughly washing the beads, cell extracts from C4-2 and LNCaP cells were added and incubated for 2 h at 4 °C. The beads were then washed, resuspended in SDS buffer, and heated to 99 °C. The SDS buffer containing the precipitated protein complex was then loaded onto a 12% SDS-PAGE gel for detection by Western blotting. Antibodies are listed in Table 3.

### Public-available data analysis

The GSE63484 ChIP-seq data was analyzed for identification of genomic LYL1 binding sites [27]. GSE137848 ChIP-seq was used for analyzing the genomic BHLHE40 binding sites [28]. GSE179684 ChIP-seq data was analyzed for finding AR binding sites with 10 nM DHT (high-T) treatment. Of note in this mentioned study 10nM DHT was used as hormone treatment for C4-2 cells, which were cultured in charcoal-stripped FBS medium. Charcoal treated serum has lower androgen level and may differ in basal level of AR regulated genes with expected lower basal expression levels. High-T, defined as 10nM DHT [29] indicates the effect of AR at SAL. GSE172205 RNA-seq data was utilized for analyzing SAL treatment (1 nM R1881) effects on expression levels of AR target genes in C4-2 cell line [11]. GSE207233 RNA-seq data was used for analyzing *LYL1* KD effects on expression levels of target genes [30]. GSE262117 RNA-seq was used for analyzing *BHLHE40* KD effects under SAL (1 nM R1881) treatment on expression levels of target genes in C4-2 cell line cultured in complete DMEM medium containing 5% untreated FBS [20]. GSE89223, GSE179321 and GSE103512 RNA-seq data were used to analyze the expression level of LYL1 in prostatectomy samples compared to adjacent tumors [31–33]. GSE148397 RNA-seq data were used to analyze the expression level of LYL1 in VCaP cells [34]. Pathway analysis was performed using PathFindR and ClusterProfiler packages [35–37]. The cytoHubba [38] from Cytoscape [39], and the STRING [40] were used to find the established network connections for LYL1. ChIP-seq visualization was performed by the IGV software [41]. Paired genes correlation was performed using the GEPIA webtool [42]. The expression level of *LYL1* was analyzed between normal tissues, primary and metastatic prostate tumors from a large cohort of PCa samples using RNA-seq data from NIH genomic data common (GDC) data portal (https://portal.gdc.cancer.gov/) with open access RNA-seq datasets from PCa patients. 52 normal samples, 539 primary, and 100 metastatic samples are included in this analysis. For the RNA-seq data of BHLHE40 KD, LYL1 KD and C4-2 at SAL, normalization and log2 fold change expression levels were calculated using DESeq2 [43] method in R. Differentially expressed genes (DEGs) with *p*-value threshold 0.05 was used as an input for pathway analysis. Using PathFindR for pathway analysis, *p* value was set at 0.05 and “Bonferroni” for adjustment method. Venn diagrams were plotted using “VennDiagram” package [44] and numbers represent overlapped genes in these data sets.

### Statistical analysis

The two-tailed Student t-test was performed for the comparison of the mean values between two groups. One-way or two-way ANOVA was used for multiple comparisons in the GraphPad Prism 8.0 software. Statistics represent the Mean of values and error bars represent SEM. For multiple comparisons, the Tukey post-hoc test was used.

## Results

### SAL induces and *BHLHE40* KD reduces the expression of LYL1 and the cell cycle inhibitor p27kip1 

**Fig. 1:**
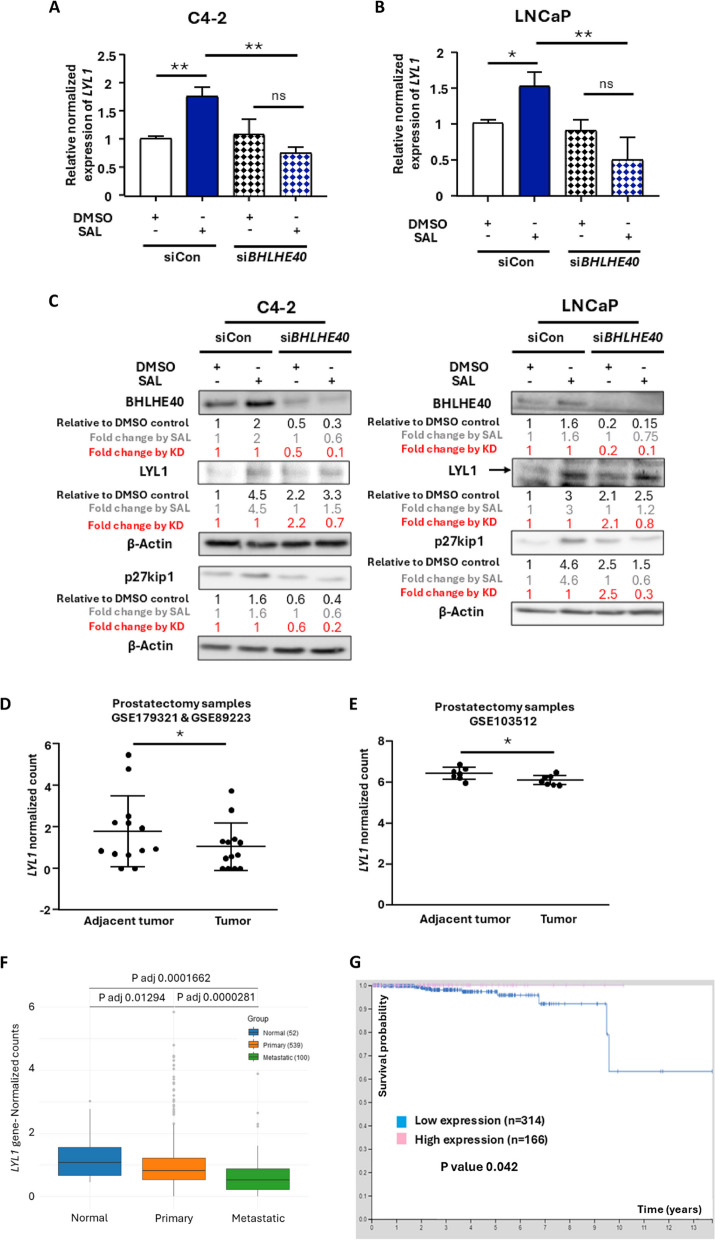
SAL enhances LYL1 expression through BHLHE40 as a tumor suppressive pathway supported by PCa datasets. **A** and **B** qRT-PCR of CRPC (C4-2) and CSPC (LNCaP) cells to detect *LYL1* mRNA levels with and without SAL treatment (1 nM R1881 (SAL), DMSO, respectively) and with and without *BHLHE40* KD (si*BHLHE40* and siCon, respectively; *n* = 3). The mRNA levels of both housekeeping genes *a-Tubulin* and *TBP* were used for normalization of expression levels. DMSO served as solvent control. **C** Western blots data from *BHLHE40* KD samples (*n* = 3) to detect *LYL1* protein level in both cell lines. p27kip1 was detected as a senescence marker. β-Actin served as loading control. The fold change by SAL is indicated as grey numbers and fold changes by each KD are indicated in red. **D** *LYL1* level in RNA-seq data from prostatectomy samples of patients comparing tumors with adjacent tissues. Reads are RPKM (Reads Per Kilobase of transcript, per Million mapped reads) normalized counts. **E** *LYL1* gene expression data were compared between tumors and adjacent tissues from patient prostatectomy samples. Gene expressions in Affymetrix data are normalized using Robust Multi-array Average (RMA) (Quantile normalization). **F** The expression level of *LYL1* in normal samples, primary and metastatic PCa tumors were extracted from a large patient cohort using RNA-seq datasets obtained from NIH genomic data common (GDC) data portal. Normalized counts are indicated as fragments per kilo base pair transcript per million reads (FPKM). The number of normal samples is 52, of primary 539, and of metastatic samples is 100. **G** Kaplan-Meier survival plot derived from the human protein atlas, comparing high and low levels of *LYL1* expression with overall survival of patients. Above 2.48 FPKM normalized count cutoff is considered as high expression of *LYL1* group and below the cut-off is defined as low expression group. Numbers in the plot represent the number of patient samples per group. *P* value = 0.042 reveals significant difference between samples for overall survival. *P* value < 0.01 = **, < 0.05 = *, ns = non-significant

Previously, we showed that SAL induces the expression of BHLHE40, which mediates SAL induced cellular senescence in both C4-2 and LNCaP PCa cell lines [20]. Here, re-analyzing our RNA-seq data from *BHLHE40* KD cells suggests that the circadian clock gene *BHLHE40* regulates the expression of LYL1 at SAL. To confirm this finding, qRT-PCR was used with RNA isolated from both C4-2 and LNCaP cells treated with DMSO, as solvent control, or SAL in combination with a pool of small interfering RNA (siRNA) mediated KD of *BHLHE40* (si*BHLHE40*). The data confirm that SAL induces the expression of *LYL1* at mRNA and enhances LYL1 protein level detected by Western blotting (Fig. 1A-C) also in non-treated serum. The KD of *BHLHE40*, reduced specifically the SAL-mediated fold induction of LYL1 (Fig. 1A-C). This indicates that BHLHE40 enhances the expression of LYL1 at SAL and is a mediator of AR signaling to upregulate LYL1.

To gain more insight into LYL1 in PCa samples, the expression of *LYL1* was compared between tumor samples and adjacent tumor tissues with datasets obtained from public available gene expression omnibus (GEO). In comparison to tumor adjacent tissues, the *LYL1* mRNA level is slightly but significantly reduced in PCa tissues (Fig. 1D and E) indicating a potential tumor suppressive activity of LYL1. In line with this, the expression level of *LYL1* was compared between normal tissues, primary and metastatic prostate tumors from a large cohort of PCa samples using RNA-seq data from NIH genomic data common (GDC) data portal. The data confirm the previous observation exhibiting a slight reduction of LYL1 in primary tumor samples compared to normal tissues and further reduction in metastatic form of PCa (Fig. 1F). These data imply that LYL1 has a tumor suppressive function in PCa. The survival plot for PCa patients from the human protein atlas shows a significant association between LYL1 expression and improved survival, it suggests that over time, patients with higher LYL1 levels have a statistically significant better outcome for overall survival. This observation supports the idea that LYL1 has a tumor suppressive role in PCa (Fig. 1G). In addition, the mRNA level of LYL1 was investigated in different PCa cell lines (figure S1A - C). In the absence of SAL low levels of LYL1 is detected in different PCa cells, while in presence of SAL, the expression of LYL1 is induced (Fig. 1A-C). R1881 at SAL, as a specific and synthetic ligand for AR, mediates cellular senescence in PCa [6] and also induces the expression level of LYL1 (Fig. 1A-C), suggesting that LYL1 is part of the AR signaling pathways and may regulate cellular senescence.

### *LYL1*KD reduces growth and induces cellular senescence through the p27kip1 cyclin dependent kinase inhibitor

**Fig. 2:**
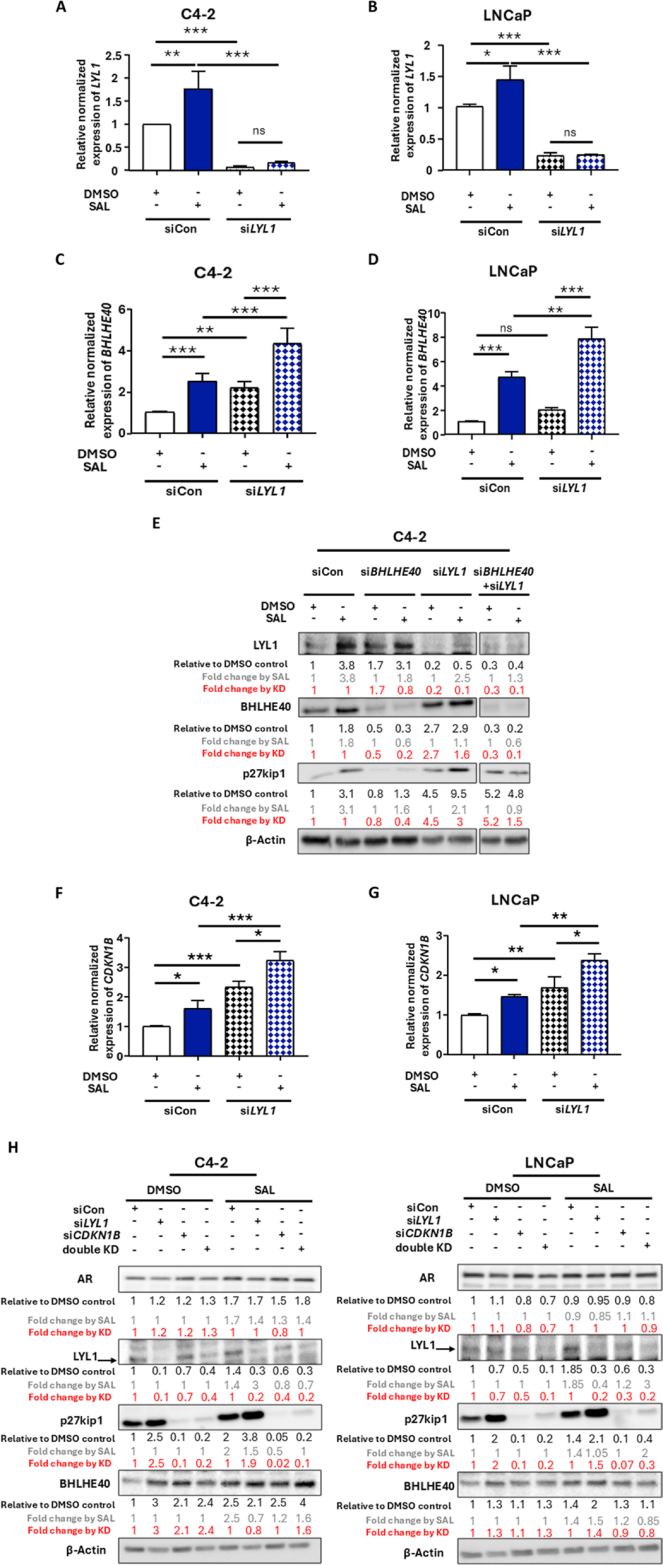
Downregulation of LYL1 induces expression of p27kip1 by upregulation of BHLHE40. **A** and **B** Detection of KD efficiency of *LYL1* by qRT-PCR in both C4-2 and LNCaP cell lines (*n* = 3). **C** and **D** Detection of mRNA level of *BHLHE40* by KD of LYL1 at SAL in both cell lines (*n* = 3). **E** Western blot analysis of cell extracts with KD of either *BHLHE40*, LYL1, or the double KD of BHLHE40 + LYL1 (*n* = 2). The fold change by SAL is indicated as gray numbers and fold changes by each KD are indicated in red. **F** and **G** mRNA level of *CDKN1B*, encoding p27kip1 as a cellular senescence marker by *LYL1* KD in treated samples compared to si control (siCon) samples in both cell lines. **H** Western blot analysis of proteins in extracts of *LYL1* KD, *CDKN1B* KD and dKD *LYL1* + *CDKN1B* samples (*n* = 2). The fold change by SAL is indicated as gray numbers and fold changes by each KD are indicated in red. *P* value < 0.001 = ***, < 0.01 = **, < 0.05 = *, ns = non-significant

To analyze the role of LYL1 in the context of SAL treatment used in BAT [7] we focused on the SAL-mediated fold inducibility of factors in the BHLHE40-LYL1 axis, which allows the identification of factors regulating SAL signaling. Interestingly, LYL1 protein level is enhanced much more by SAL compared to the mRNA level. This may suggest that *LYL1* mRNA may not reflect LYL1 protein level and that LYL1 protein is stabilized by SAL treatment. To investigate the role of LYL1 in growth and induction of cellular senescence in PCa cells, first the levels of p27kip1 were analyzed by SAL treatment and by the KD of *BHLHE40*. The data suggest an upregulation of the cell cycle inhibitor and senescence inducer p27kip1 [45] by SAL and a blunted induction via the KD of *BHLHE40* in both cell lines (Fig. 1C). Next, siRNA targeting *LYL1* shows high KD efficiency (Fig. 2A and B). Interestingly, si*LYL1* induces BHLHE40 expression in both PCa cell lines (Fig. 2C-E) and suggests a negative feedback loop between LYL1 and BHLHE40 by which BHLHE40 enhances LYL1, which in turn suppresses BHLHE40 expression. Focusing on SAL treatment, our data suggest that in the presence of SAL, BHLHE40 induces LYL1 expression (Figs. 1C and 2E), which may be due to the AR-BHLHE40 protein-protein interaction described earlier [20]. Focusing on the absence of SAL treatment, interestingly, the KD of *BHLHE40* enhances LYL1 protein level (Figs. 1C and 2E), however, without affecting the *LYL1* mRNA level (Fig. 1A, B). This suggests that BHLHE40 regulates LYL1 protein level at post-transcriptional level in the absence of SAL. The KD of *LYL1* enhanced p27kip1 level, which was further enhanced by SAL treatment (Fig. 2E) indicating that the potent KD of *LYL1* induces cellular senescence, which might derive from the augmented level of BHLHE40 by the *LYL1* KD. Therefore, we hypothesized that the potent induction of p27kip1 is mediated through upregulation of BHLHE40 or downregulation of LYL1. To verify our hypothesis, rescue experiments were performed by employing a double knockdown (dKD) of both *LYL1* and *BHLHE40* (Fig. 2E). The rescue experiments confirmed the hypothesis by indicating that the induction of p27kip1 by the KD of *LYL1* at SAL is mediated by increased level of BHLHE40 since p27kip1 level is suppressed when *BHLHE40* is also knocked down (Fig. 2E). The SAL-mediated fold induction of protein levels, comparing DMSO with SAL, of BHLHE40 and p27kip1 by the *LYL1* KD is weaker compared to siControl, indicating that LYL1 regulates inducibility of these factors by SAL. We observed a complete loss of inducibility of p27kip1 by SAL in the dKD cells (Fig. 2E). Interestingly, the basal levels of p27kip1 are higher in the dKD compared to the DMSO control suggesting that both transcription factors LYL1 and BHLHE40 regulate indirectly the protein levels of p27kip1. Of note, p27kip1 protein levels are mainly controlled by protein degradation via the ubiquitin-proteasome pathway [46].

It was previously found that LYL1 KO mice exhibit an enhanced apoptosis rate [47]. To investigate the effect of *LYL1* KD in induction of apoptosis, c-PARP as a marker of cell apoptosis was analyzed (figure S1). Since no cleaved-PARP was detected after KD of *LYL1*, it indicates that downregulation of LYL1 is not measurably mediating apoptosis in PCa cell lines. To address the question whether the *LYL1* KD interferes with AR mediated transactivation, the expression levels of direct AR target genes were analyzed in *LYL1* KD cells. The data suggest that there are no significant changes of *FKBP5* and *KLK3* mRNA levels upon *LYL1* KD (figure S2A - D) indicating that LYL1 is downstream of AR signaling. Accordingly, the AR protein level is not detectably changed by the KD of *LYL1* (Fig. 2H). Gene correlation study by using datasets of prostate adenocarcinoma (PRAD) from TCGA and of normal prostate samples from GTEx (Genotype-Tissue Expression) from GEPIA for *LYL1* and each of the analyzed known AR direct target genes revealed a negative correlation with the *LYL1* expression (figure S2E - I). The correlation analyses indicate that AR signaling in tumor is associated with reduced LYL1 expression in PCa. However, the correlation analyses do not reflect any hormone or SAL treatments in patients. It has been shown that SAL, compared to physiological androgen levels, significantly alters the transcriptome landscape, not only induces but also represses gene expression, and induces cellular senescence as a tumor suppressive pathway [15, 20, 48]. The SAL mediated effects are AR dependent since on one hand AR negative cells treated with SAL do not respond and on the other hand re-expression of AR mediates cellular senescence by SAL [6].

To address the possibility that LYL1 may co-regulate SAL induced cellular senescence, the expression of *CDKN1B* gene encoding p27kip1 was analyzed. As expected, *LYL1* KD induces significantly both mRNA and protein levels of p27kip1 in both C4-2 and LNCaP cells (Fig. 2E-H).

**Fig. 3:**
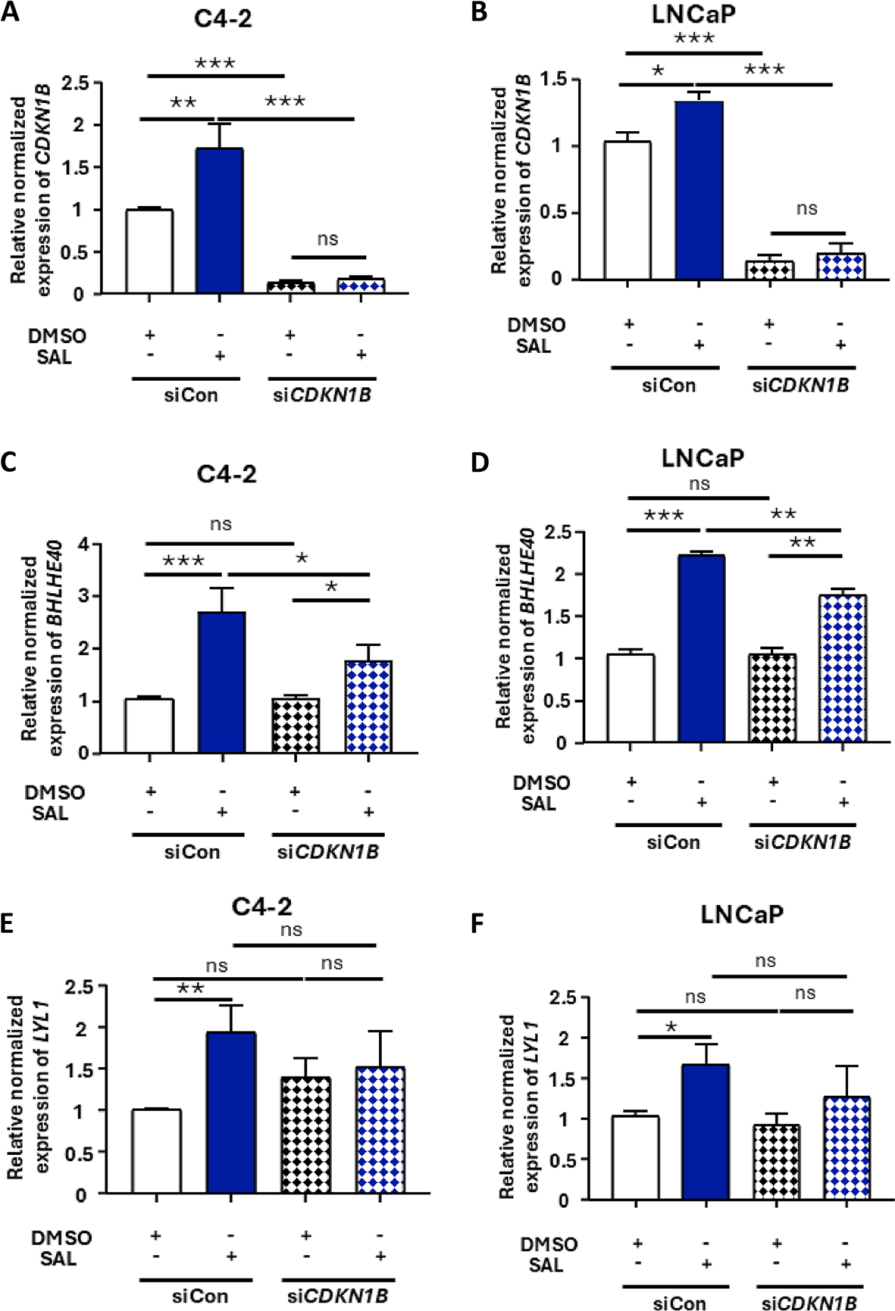
A novel feedback circuit: p27kip1 KD reduces *BHLHE40* and *LYL1* levels. **A** and **B** Detection of *CDKN1B* mRNA encoding p27kip1 by qRT-PCR using siRNA mediated KD in with and without SAL treatment in both cell lines (*n *= 3). **C** and **D** *BHLHE40* mRNA was analyzed via qRT-PCR in C4-2 and LNCaP cells in control (siCon) and KD of *CDKN1B*. The *CDKN1B* KD significantly reduces *BHLHE40* mRNA level specifically at SAL (*n* = 3). **E** and **F **The mRNA levels of *LYL1* were analyzed via qRT-PCR with and without KD of *CDKN1B* in C4-2 and LNCaP (*n* = 3). *P* value < 0.001 = ***, < 0.01 = **, < 0.05 = *, ns = non-significant

To address the question whether there is also feedback signaling by p27kip1, the KD of p27kip1 (si*CDKN1B*) were performed in both C4-2 and LNCaP cells (Figs. 2H and 3A-B). An efficient KD of p27kip1 was detected and resulted in reduction of the SAL mediated fold induction of both BHLHE40 and LYL1 at protein level while that the reduced fold induction of *LYL1* mRNA level is less pronounced. The KD of p27kip1 reduces also the fold SAL mediated induction of BHLHE40 (Figs. 2H and 3C-F). It is important to note that the lower effect of p27Kip1 KD on BHLHE40 protein level might be due to the fact that BHLHE40 protein may have a longer half-life and slow degradation, perhaps through protein-protein interactions with AR under SAL treatment [20]. Since on one hand the *LYL1* KD enhances p27kip1 and on the other hand the KD of p27kip1 leads to reduction of LYL1 at protein level, it suggests the identification of another feedback loop within this circuit LYL1 represses p27kip1 whereas p27kip1 enhances LYL1. The data suggest that the fold inducibility by SAL is controlled by BHLHE40, LYL1 and p27kip1 within crosstalk.

### *LYL1* KD induces cellular senescence and resulted in growth inhibition of both CSPC and CRPC

**Fig. 4:**
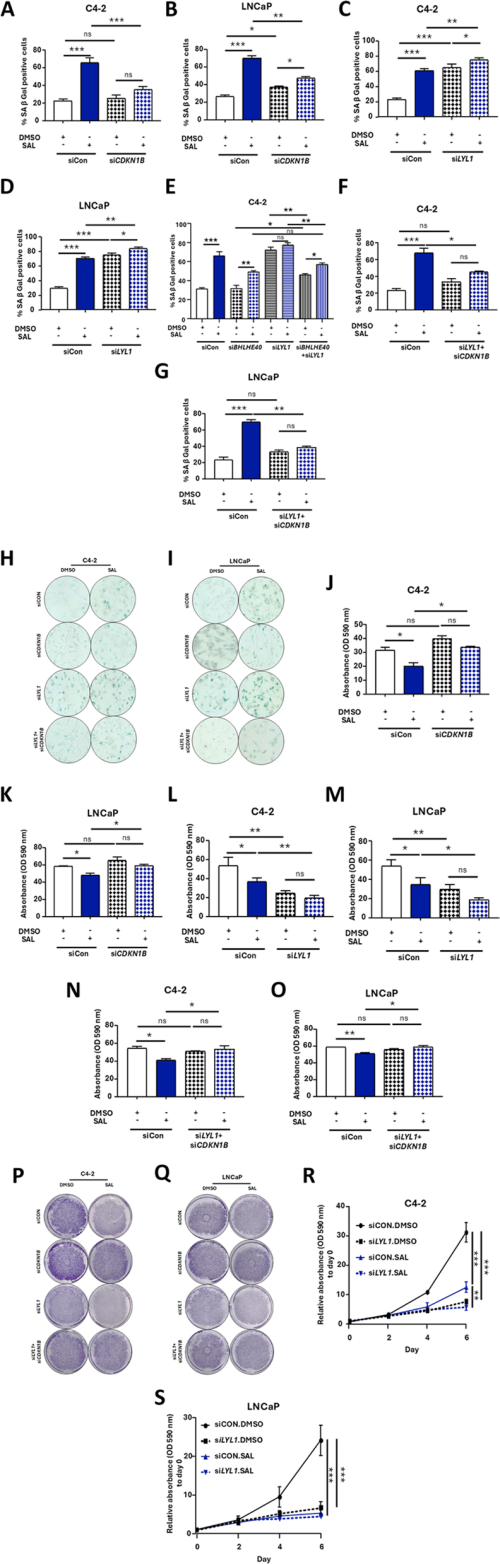
Cellular senescence induction and growth inhibition by SAL is mediated by increased BHLHE40 and p27kip1. **A** and **B** SA β-Gal activity for SAL-treated and *CDKN1B* KD samples at SAL (*n* = 3). **C** and **D** SA β-Gal activity for SAL-treated and *LYL1* KD samples after KD in both cell lines (*n* = 3). **E** SA β-Gal activity for SAL-treated, *LYL1* KD, *BHLHE40* KD and *BHLHE40* + *LYL1* dKD samples. **F** and **G** SA β-Gal activity for dKD (*LYL1*+*CDKN1B*) samples in both cell lines (C4-2 *n* = 3, LNCaP *n* = 2). **H** and **I** Representative cellular senescence pictures of both cell lines. **J** and **K** Crystal violet staining for *CDKN1B* KD samples to analyze cell growth at SAL (*n* = 3). **L** and **M** Crystal violet staining 3 days after treatments analyzing growth of SAL-treated and *LYL1* KD samples in both cell lines (*n* = 3). **N** and **O** Crystal violet staining for SAL-treated and dKD (*LYL1*+*CDKN1B*) samples in both cell lines (C4-2 *n* = 3, LNCaP *n* = 2). **P** and **Q** Representative pictures for crystal violet staining of both cell lines. **R** and **S** Growth curve analyzed by crystal violet up to 6 days for KD of *LYL1* in both cell line (*n* = 2). *P* value < 0.001 = ***, < 0.01 = **, < 0.05 = *, ns = non-significant

To investigate whether LYL1 mediates cellular senescence by SAL and whether LYL1 and p27kip1 are acting together in the same signaling axis in order to mediate cellular senescence, the dKD of *LYL1* and *CDKN1B* was performed in both cell lines (Fig. 2H and S3A-D). As expected, the treatment of both cell lines with SAL enhanced the SA β-galactosidase activity (SA-β Gal) suggesting increased cellular senescence being in accordance with reduced growth of cells (Fig. 4A, B and J, K). The KD of p27kip1 did not change the basal senescence level but resulted in reduced level of cellular senescence by SAL and accordingly induced cell growth in both cell lines (Fig. 4A, B and J, K). These data strongly support the notion that p27kip1 is one key factor for growth arrest by SAL-induced cellular senescence of these PCa cells. Representative pictures for cellular senescence and growth levels are depicted in (Fig. 4H, I and P, Q, respectively).

In line with the upregulation of p27kip1, the KD of *LYL1* enhances the level of cellular senescence and reduces the cell population (Fig. 4C, D and L, M). Representative pictures for cellular senescence and growth levels are depicted in (Fig. 4H, I and P, Q) respectively. Furthermore, growth curve analysis up to 6 days after treatments for *LYL1* KD shows that SAL reduces significantly cell growth, which is observed already at day 2, whereas cells with *LYL1* KD show further inhibition of growth in both cell lines (Fig. 4R and S). These results are in agreement with cell growth inhibition 72 h after treatment.

To confirm that the enhanced senescence levels are due to augmented BHLHE40 level via the KD of *LYL1*, rescue experiments were performed employing the dKD of *BHLHE40* and *LYL1*. Reducing the BHLHE40 level by KD resulted in suppressed cellular senescence in the dKD cells compared to *LYL1* KD confirming that *LYL1* KD mediates cellular senescence through upregulation of BHLHE40, which is in agreement with the detected p27kip1 levels (Fig. 4E and S4). This further confirms a negative regulatory loop between BHLHE40 and LYL1.

The induction of cellular senescence by single KD of *LYL1* was rescued after dKD of *LYL1* and *CDKN1B* in both cell lines (Fig. 4F and G). In line with this, also the growth of both cell lines is restored by SAL treatment (Fig. 4N and O) suggesting that LYL1 mediates in part cellular senescence through induction of BHLHE40 and p27kip1. Representative pictures of cellular senescence and growth inhibition after *LYL1* and *CDKN1B* dKD are depicted in (Fig. 4H, I and P, Q) respectively. These results together suggest that diminishing LYL1 alleviates its inhibitory effects and driving cells towards cellular senescence through p27kip1, which is the first report of the p27kip1 to mediate SAL-induced cellular senescence in PCa.

### *E2F1* as a cell cycle mediator is reduced by *LYL1* KD and induced by *CDKN1B* KD

**Fig. 5:**
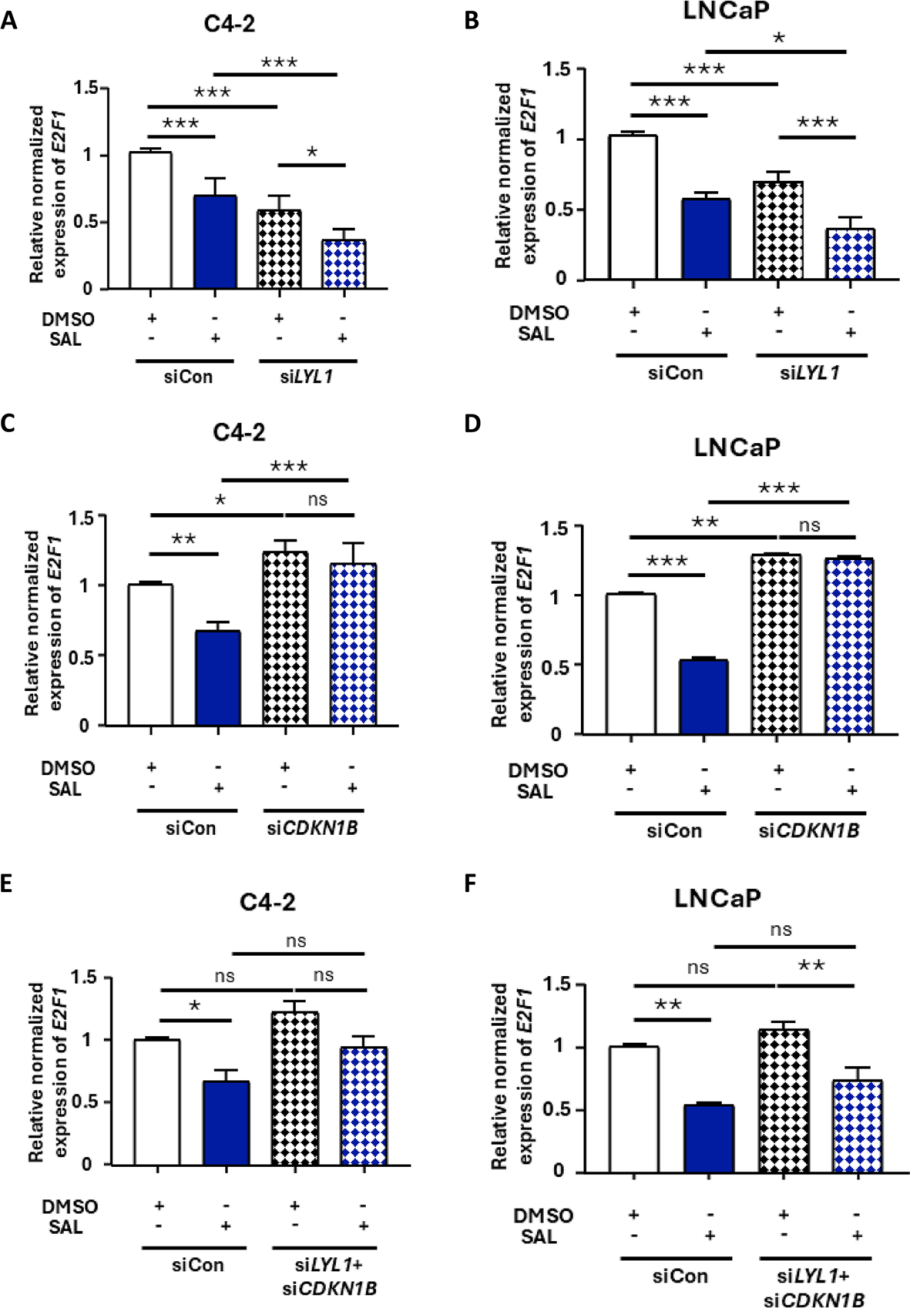
*E2F1* levels confirm inhibitory effects of *LYL1* KD on cell growth. Analyzing the expression level of *E2F1* by qRT-PCR. **A** and **B** mRNA level of *E2F1* in *LYL1* KD samples (*n* = 3). **C** and **D** *E2F1* mRNA level after *CDKN1B* KD (*n* = 3). **E** and **F** mRNA level of *E2F1* in dKD (*LYL1*+*CDKN1B*) samples in both C4-2 and LNCaP cell lines (C4-2 *n* = 3, LNCaP *n* = 2). *P* value < 0.001 = ***, < 0.01 = **, < 0.05 = *, ns = non-significant

E2F1, is a critical cell cycle promoting factor [49]. Therefore, as KD of *LYL1* showed induction of cellular senescence the change of *E2F1* expression was analyzed. SAL reduced *E2F1* expression level that confirms growth inhibitory effect of SAL in PCa cell lines (Fig. 5A and B). A further significant reduction of *E2F1* is detectable after KD of *LYL1* in both cell lines (Fig. 5A and B). These data are in agreement with the induction of cellular senescence and reduction of cell growth by *LYL1* KD. Accordingly, significant induction in the mRNA level of *E2F1* by KD of *CDKN1B* is detected (Fig. 5C and D). Notably, the dKD of *LYL1* and *CDKN1B* rescued the effect of changed *E2F1* level since the KD of *CDKN1B* reversed the reduction of *E2F1* by *LYL1* KD to the basal level (Fig. 5E and F). This indicates that LYL1 and p27kip1 are acting in concert within the same axis to regulate cell growth and cellular senescence induced by SAL.

### *LYL1* KD induces cellular senescence and reduces Ki67 proliferation marker in CRPC 3D tumor spheroids

**Fig. 6:**
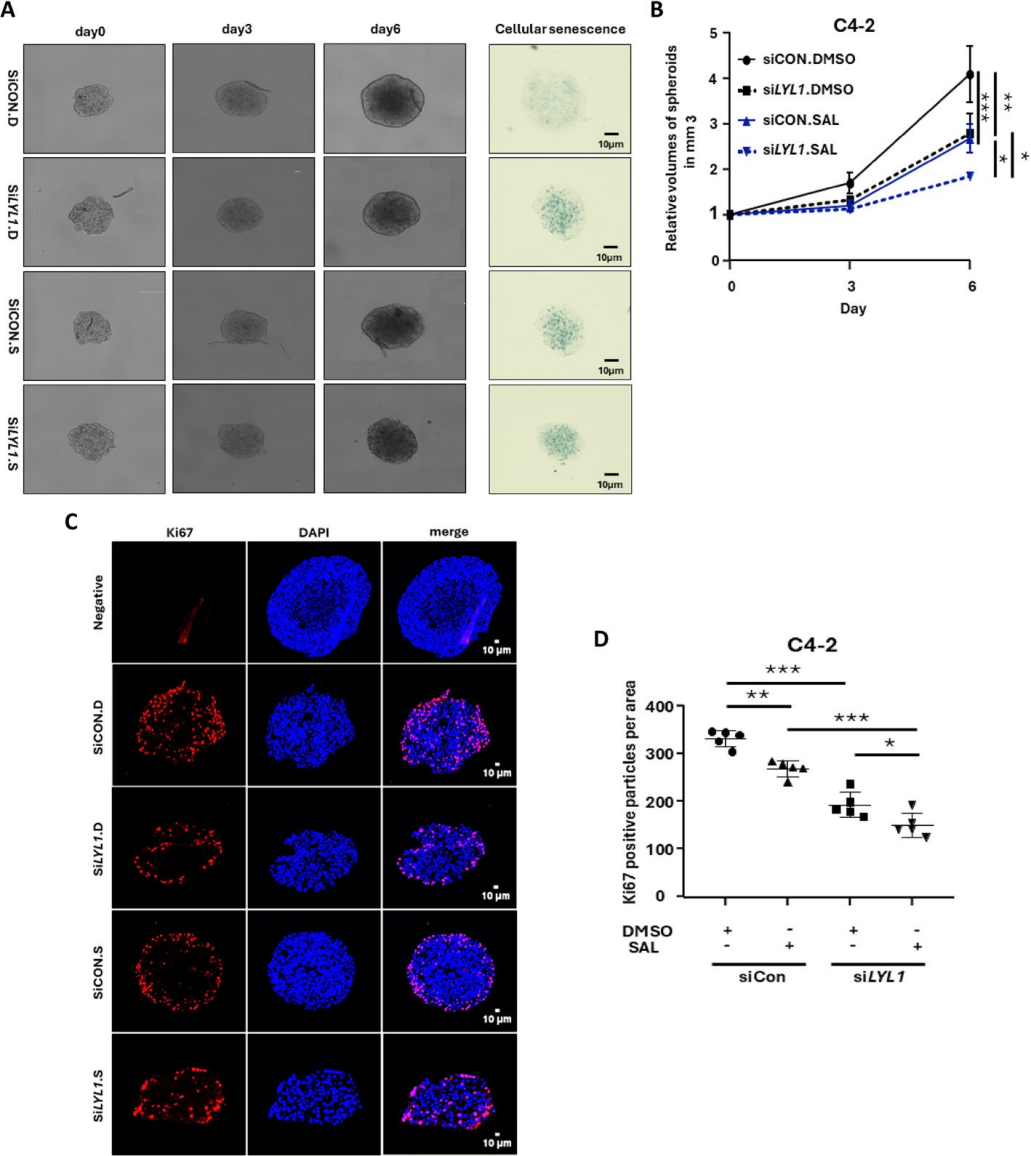
*LYL1* KD reduces cellular senescence and reduces Ki67 proliferation marker in 3D tumor spheroid model. **A** Representative pictures for the volume of the spheroids treated with or without SAL and *LYL1* KD (*n* = 2 biological replicates). **B** graph represents tumor spheroids volume for 6 days after treatments indicated as relative to day 0, which is set arbitrary as 1. **C** Immunofluorescence staining for Ki67 in spheroids treated with or without SAL and *LYL1* KD (*n* = 2). **D** Quantification for Ki67 positive staining (*n* = 2). *P* value < 0.001 = ***, <0.01 = **, <0.05 = *

The 3D tumor spheroid model surpasses 2D cell cultures in terms of complexity and drug delivery [50]. 3D spheroids were formed from parental C4-2 and *LYL1* KD cells using ultra-low attachment plates following the previously described method [11]. In line with the findings by the 2D adherent cultures, SAL reduces spheroid volumes (figure 6A and B). The *LYL1* KD leads to a further reduction of spheroid volume in both DMSO and SAL-treated samples (figure 6A and B). SA β-Gal activity staining of the tumor spheroid slices revealed an increase in staining after treatment with SAL in control samples. In spheroids generated from *LYL1* KD cells, the induction of SA β-Gal activity staining is higher compared to control samples (figure 6A). Immunohistochemistry analysis of the proliferation marker Ki67 indicates an increase in the number of Ki67-positive staining in control (DMSO)-treated spheroid slices compared to SAL-treated ones (figure 6C and D), supporting a tumor suppressive mechanism by SAL. This aligns with the increased levels of senescent cells in SAL-treated samples. The *LYL1* KD resulted also in less Ki67-positive staining compared to control DMSO, with an even further reduction under SAL treatment (Fig. 6C and D). These findings are consistent with results from 2D adherent cultures, providing further evidence that the KD of *LYL1* induces cellular senescence in PCa cells and reduces growth in both 2D and 3D models.

### LYL1 interacts with BHLHE40 with a large overlap of genes leading to a transcriptome landscape with several cellular senescence related pathways

**Fig. 7:**
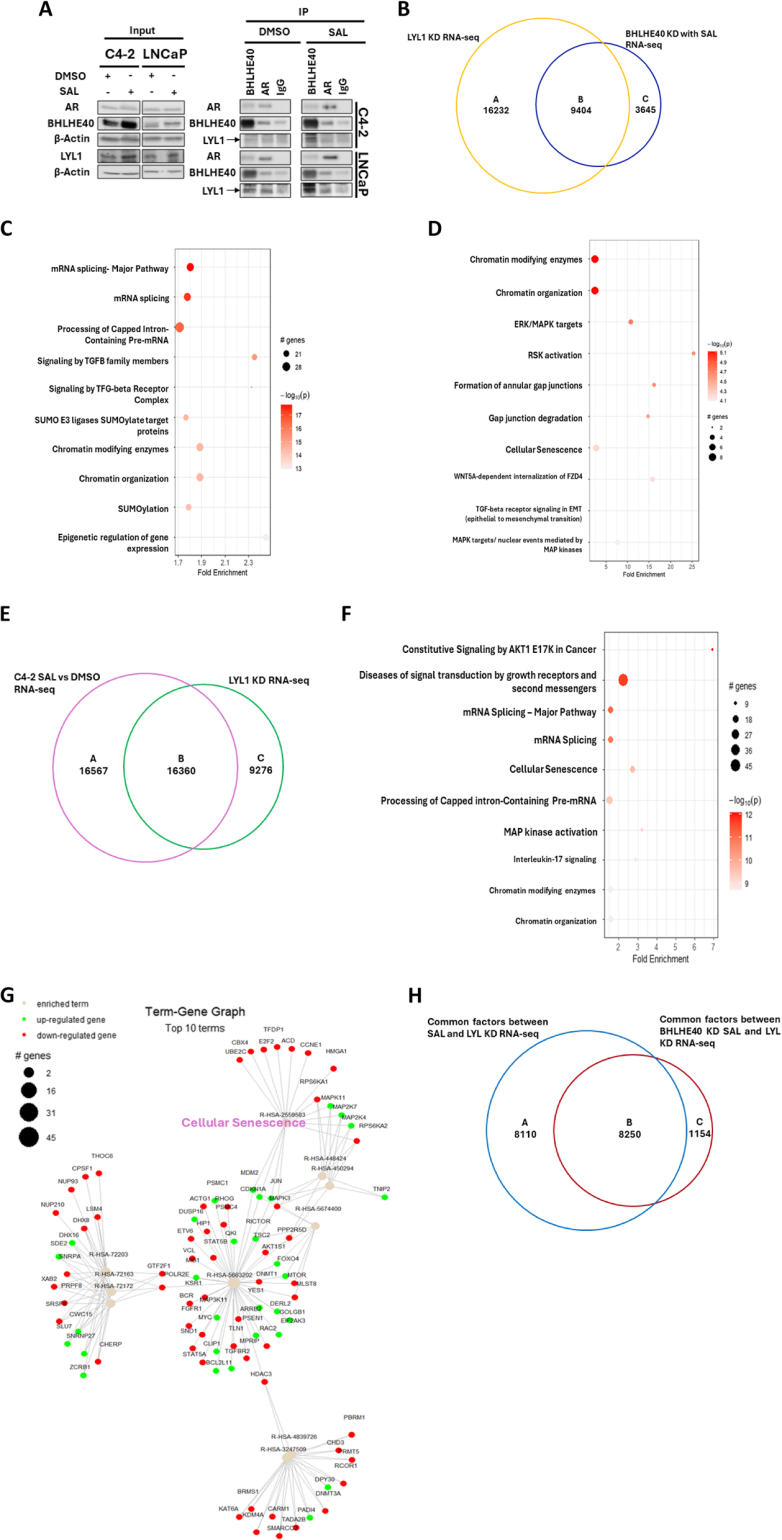
BHLHE40 interacts with LYL1 with larger overlap of genes between BHLHE40 and LYL1. **A** Co-IP of AR and BHLHE40 were performed with AR and BHLHE40 transcription factors in both LNCaP and C4-2 cell lines. Western blot indicates the immunoprecipitated AR, BHLHE40 and LYL1. **B** Venn diagram depicts the number of overlapping factors between *LYL1* KD and *BHLHE40* KD transcriptomes. **C** Pathway analysis of significantly expressed common genes was performed by pathfindR. **D** Pathway analysis for *LYL1* KD specific factors. **E** Venn diagram indicates the number of overlapping factors between *LYL1* KD and SAL transcriptomes from C4-2 cell line. **F** Pathway analysis for common significant genes regulated by *LYL1* and SAL treatment performed by pathfindR. **G** Top 10 pathways interactions from panel F were analyzed with PathfindR by considering the log2FC of LYL1 KD DEGs for showing the expression regulation. Pathways are labeled with Reactome ID. **H** Venn diagram shows the number of common factors from panel B and E

To analyze the potential interaction between LYL1 and AR or BHLHE40, Co-IP experiments were performed for AR and BHLHE40 factors in both cell lines. LYL1 was detected in the immunoprecipitated BHLHE40 samples indicating a protein-protein complex between BHLHE40 and LYL1 (Fig. 7A). Interestingly, the interaction of LYL1 with BHLHE40 was enhanced using cells that were treated with SAL (Fig. 7A). This suggests that the interaction between LYL1 and BHLHE40 is modulated by AR. While in C4-2 cells we could not detect LYL1 above background in the immunoprecipitated material of AR, enhanced LYL1 was co-immunoprecipitated with AR in LNCaP cells (Fig. 7A) suggesting that in LNCaP cells LYL1 interacts with BHLHE40 and the AR.

Therefore, we hypothesized that LYL1 and BHLHE40 have in common a set of DEGs resulting in pathways that regulate cellular senescence at SAL. For that purpose, we analyzed transcriptome data of LYL1 with that of BHLHE40 and SAL. The RNA-seq datasets were downloaded from GEO derived from *LYL1* KD and compared the transcriptomes with our previously published RNA-seq data derived from *BHLHE40* KD at SAL. The bioinformatic analyses suggest that there is indeed a large overlap of genes between LYL1 and BHLHE40 (Fig. 7B and supplementary excel file). Pathway analysis for these significantly expressed common genes with cutoff P value 0.05 including 573 genes revealed a set of cellular senescence-linked pathways including “chromatin organization”, “cellular senescence”, and “TGF-β signaling pathways” (Fig. 7C; Table 4) which highlight the potential role of LYL1 and BHLHE40 transcription factors in cellular senescence regulation.

**Table 4:** Pathways for common factors between *BHLHE40* at SAL and *LYL1* KD

	*P value*
Mitochondrial translation	1.66E-06
**Cellular senescence**	**1.65E-06**
Oxidative stress induced senescence	9.74E-05
Estrogen-dependent gene expression	8.50E-06
Repression of WNT target genes	2.15E-05
Signaling by NOTCH	0.000881
Synthesis of DNA	0.000921
Cellular response to chemical stress	0.005142
Oncogenic MAPK signaling	0.034401
G1/S DNA damage checkpoints	0.021354

Moreover, pathway analysis was applied for specific genes derived from LYL1 KD DEGs with cutoff P value 0.05 including 382 genes (Fig. 7B and supplementary excel file). Results revealed pathways including “cell cycle regulations”, “ERK/MAPK targets”, and “Signaling by NTRKs” in addition to those from common factors including cellular senescence pathways (Fig. 7D; Table 5) suggesting that LYL1 transcription factor itself regulates cellular senescence.

**Table 5:** Pathways for specific genes from *LYL1* KD RNA-seq

	*P value*
Signaling by TGFB family members	0.000393
Signaling by NTRKs	0.000704
Toll Like Receptor 3 (TLR3) cascade	0.002961
TNF signaling	0.007338
Signaling by TGF-beta receptor complex	0.018245
G1/S transition	0.033945
TP53 regulates transcription of genes involved in G1 cell cycle arrest	0.035669

Also, the transcriptome data of C4-2 cells treated with SAL was used to analyze the overlap with that of RNA-seq data derived from *LYL1* KD to identify potential genes that are specially regulated by both SAL and LYL1. Of note, again a large overlap of genes was detected between SAL and *LYL1* KD samples (Fig. 7E and supplementary excel file). Pathway analysis of significantly expressed genes with cutoff P value 0.05 including 899 genes from this overlap set revealed “cellular senescence” within the top 10 significant pathways (Fig. 7F). These analyses further strengthen the notion that LYL1 is part of SAL-induced cellular senescence.

Network interactions among the top pathways were performed and expression direction of involved genes in each pathway is depicted by considering the log2FC of DEGs from *LYL1* KD RNA-seq (Fig. 7G). The network interactions plot revealed that identified genes in the cellular senescence pathway are linked to “interleukin-17 signaling”, “MAP kinase activation”, “Constitutive Signaling by AKT1 E17K in Cancer “and “Diseases of signal transduction by growth factor receptors and second messengers” pathways.

To identify those factors that are commonly regulated by SAL, LYL1 and BHLHE40 the common factors that are depicted in the Venn diagram (Fig. 7B) and those common factors indicated in the Venn diagram (Fig. 7E) were further analyzed (Fig. 7H and supplementary excel file). Interestingly, a large overlap of gene sets has been identified that supports the notion that LYL1 and BHLHE40 are important transcription factors within AR signaling. Of note, cellular senescence pathways emerged by pathway analysis for significantly expressed genes with cutoff *P* value 0.05 including 546 genes from these common genes (Table 6) suggesting a functional relationship of LYL1 and BHLHE40 in SAL-induced cellular senescence.

**Table 6:** Pathways for common genes (panel B in figure 7H)

	*P value*
**Signaling by TGF-beta Receptor Complex**	3.19E-08
Estrogen-dependent gene expression	2.06E-05
Signaling by nuclear receptors	2.27E-05
**Cellular senescence**	4.05E-05
**DNA repair**	0.000252
Nuclear receptor transcription pathway	0.000352
Growth hormone receptor signaling	0.004723
Cellular response to chemical stress	0.020234
**Senescence-Associated Secretory Phenotype (SASP)**	0.043275

**Fig. 8:**
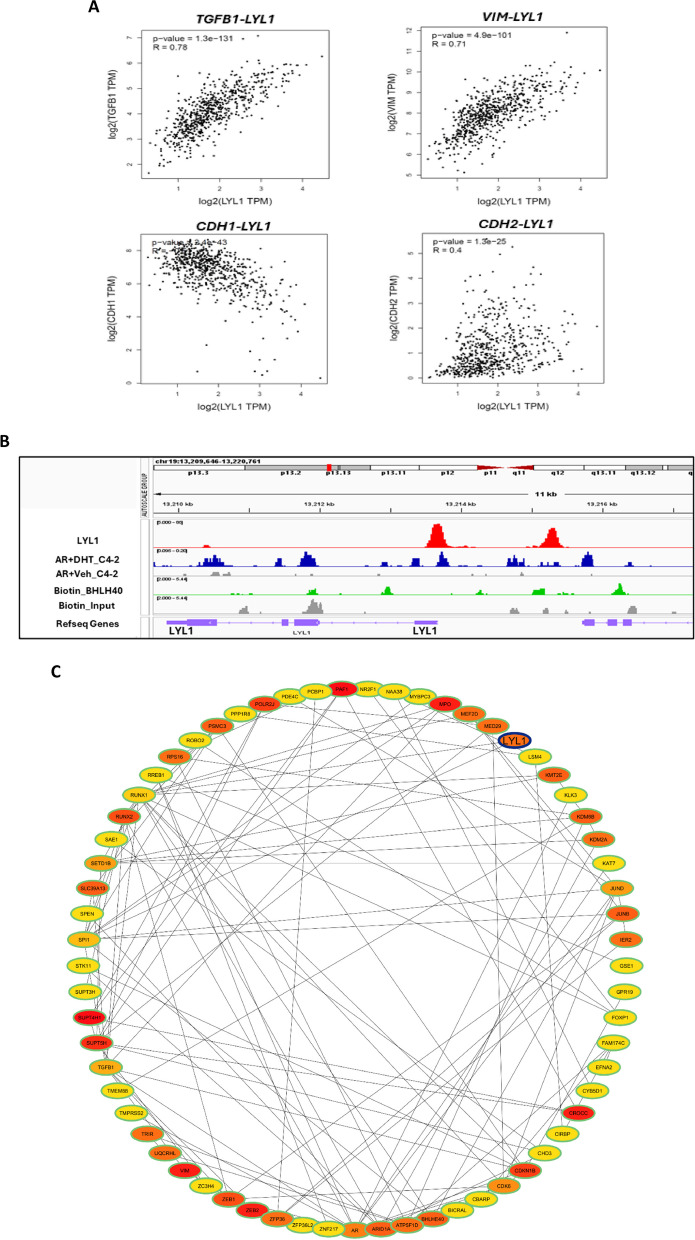
AR, BHLHE40 and LYL1 show DNA recruitments on the LYL1 gene region. **A** gene correlation (spearman) study between LYL1 and each of EMT markers using GEPIA. **B** ChIP-seq data were analyzed for recruitment of AR and BHLHE40 to the LYL1 gene with IGV software for visualization. hg19 was utilized as a reference genome in IGV for peak visualization. **C** Network interaction analysis of LYL1, BHLHE40, AR transcription factors and *CDKN1B* along with EMT related factors. STRING was used for network interactions and Cytoscape was used for making plot

In addition to these mentioned pathways, “TGF-β signaling pathways” appeared multiplied in the pathway analysis. TGF-β signaling is linked to epithelial-mesenchymal transition [51, 52], Therefore, gene correlation analyses of *LYL1* with *TGFB*, encoding TGF-β, and markers of EMT including *CDH1*, encoding E-Cadherin, *CDH2*, encoding N-Cadherin, and *VIM*, encoding Vimentin, were performed by using datasets of PRAD from TCGA and of normal prostate samples from GTEx in GEPIA. The data suggest that there is a strong correlation between *LYL1* with each of the EMT factors (Fig. 8A).

Next, we addressed the question whether *LYL1* is purely associated with EMT markers expression or has a causative role in the regulation of the expression of vimentin, E- and N-cadherins. For that purpose, using KD of *LYL1*, the mRNA expression was analyzed by qRT-PCR in both C4-2 and LNCaP cell lines. The results suggest upregulation of these EMT markers and suggests that in these PCa cell lines *LYL1* negatively regulates both epithelial (*CDH1*) and mesenchymal markers (*CDH2*) with no significant effects for vimentin as mesenchymal marker (figure S5). Thus, LYL1 expression is associated with some EMT markers in PCa. The lack of LYL1 controlled vimentin expression is in line with the lack of SAL regulated vimentin expression in C4-2 cells and a distinct response of epithelial-mesenchymal factors [53].

Next, based on the protein interaction the chromatin recruitment of AR, BHLHE40 and LYL1 transcription factors were explored. Publicly available ChIP-seq data of LYL1, AR at SAL, and BHLHE40 were investigated. IGV software visualized that both LYL1 and AR are recruited to a similar region in the *LYL1* gene (Fig. 8B). Also, other binding sites for AR are detected in the *LYL1* gene. These data suggest that LYL1 is a direct target of AR and LYL1 and imply that it can also autoregulate its own expression (Fig. 8B). In addition to AR and LYL1, BHLHE40 ChIP-seq showed chromatin recruitment for BHLHE40 to the *LYL1* gene, which suggests that LYL1 is regulated by BHLHE40 as well (Fig. 8B). Together these data represent that in addition to autoregulation of LYL1, AR and BHLHE40 might regulate directly the *LYL1* gene.

To investigate the possible network connection of LYL1 with BHLHE40, AR, p27kip1 and EMT markers, cytoHubba from Cytoscape, and STRING webtool were utilized (Fig. 8C). Ranking is based on MCC method and colors represent importance of the nodes. The data suggests that LYL1 interacts with factors including EMT markers being at a higher rank compared to other factors and is in agreement with correlation analysis data obtained from GEPIA.

## Discussion

PCa is a public health problem mostly reported in developed countries, which affects large populations in western countries [1]. It is the fifth most common male tumors in the world and the second most common cause of cancer death in men [1]. The AR has a pivotal role in PCa, including castration resistant PCa [54, 55]. Interestingly, the AR has both tumor suppressive and oncogenic function [2]. It has been shown that SAL mediates inhibitory effects on tumor growth, especially in PCa cells that grow in low androgen levels [6]. As a cellular response, SAL induces cellular senescence in an AR dependent manner in both CSPC and CRPC [11, 15]. Also, cellular senescence is induced by SAL in C4-2 xenografted mice and in prostatectomy tissues treated ex vivo with SAL [20]. Cellular senescence defines as a stable arrest of cells in G0/G1 phase which is considered as a mechanism against cancer progression [3]. Previously we showed that SAL induces the expression of BHLHE40, a basic Helix Loop Helix transcription factor, that mediates cellular senescence in PCa cell lines and we linked functionally the clock gene BHLHE40 to androgen signaling [20].

It has been found that higher expression levels of BHLHE40 is correlated with shorter disease-free survival and overall survival in AML patients [56]. Here we show that LYL1 is part of the BHLHE40 signaling that mediates SAL-induced cellular senescence. LYL1 (Lymphoblastic leukemia 1) is a member of class II basic Helix Loop Helix (bHLH) proteins, which forms heterodimer with class I and functions as a transcription factor [57, 58]. Initially, LYL1 was found in T-ALL cells [59]. It was shown before that LYL1 is necessary for assembling and making AETFC (AML1-ETO Transcription Factor Complex) in AML. Notably, the LYL1-containing AETFC preferentially binds to active enhancers and mediates gene activation. Another study has shown that LYL1 is regulated by super-enhancers and it is promoting cell growth and survival of AML [23].

Most of the studies analyzing LYL1 function were performed in different types of blood cancer including AML. To the best of our knowledge there is no study regarding the function of LYL1 in cellular senescence regulation and in PCa. Here, we investigated the function of LYL1 in regulation of cellular senescence in PCa cell lines induced by SAL. We analyzed RNA-seq datasets from prostatectomy samples and adjacent tissues and also included the expression of a large sample cohort of 52 normal prostate, 539 primary prostate tumors and 100 metastatic prostate tumors. The obtained data revealed a reduction of LYL1 in tumor samples and further reduction in metastatic form of cancer suggesting that LYL1 might have tumor suppressive activity in PCa. Our data suggests that SAL induces the expression of both BHLHE40 and LYL1 in PCa cell lines. The KD of *BHLHE40* reduces the expression of LYL1 indicating that BHLHE40 positively regulates LYL1, and SAL may induce LYL1 through upregulation of BHLHE40. On the other hand, the KD of *LYL1* via a feedback loop induces the expression of BHLHE40.

Another interesting novel feedback loop is identified here involving p27kip1, a cyclin dependent kinase inhibitor that inhibits CDK2 and CDK4 [60]. It has been shown that p27kip1 is involved in many cell processes including cellular senescence [45, 61–63]. The KD of *LYL1* potently induces cellular senescence by inducing the expression level of p27kip1. The KD of p27kip1 reduces cellular senescence indicating that p27kip1 is part of LYL1 signaling. In line with this, KD of *BHLHE40* reduces cellular senescence and the levels of p27kip1. The dKD of *LYL1* and *BHLHE40* rescued that effect by reducing the induction of p27kip1. This suggests that p27kip1 is acting within the signaling axis of both BHLHE40 and LYL1 to mediate cellular senescence. As the basal levels of p27kip1 are still higher in the dKD compared to the DMSO control it suggests that both transcription factors LYL1 and BHLHE40 regulate indirectly and perhaps, in addition, independently the protein levels of p27kip1. Of note, p27kip1 protein levels are mainly controlled by protein degradation via the ubiquitin-proteasome pathway indicating other factors also control its protein level [46].

The KD of p27kip1 inhibits cellular senescence and confirmed its role in induction of cellular senescence being to our knowledge the first report that p27kip1 expression is linked to a clock gene and androgen signaling. Another novel negative feedback loop is identified here between LYL1 and p27kip1, which was confirmed by the rescue effect of p27kip1 on cellular senescence. This circuit includes that p27kip1 induces LYL1 while LYL1 inhibits p27kip1. The data further suggests that LYL1 mediates in part cellular senescence through induction of BHLHE40 and p27kip1.

Interestingly, the bioinformatic analysis revealed a large overlap between BHLHE40, LYL1 and SAL transcriptomes indicating that these two helix-loop-helix factors work in concert within the AR signaling in PCa cells. Multiple cellular senescence pathways emerged by pathway analysis for common significantly expressed genes. Correlation analysis for EMT markers with LYL1 showed high correlation of co-expression between LYL1 and EMT markers. The KD of *LYL1* suggests that this observation is not based on a causative correlation but a clinical association. Taken together as KD of *BHLHE40*, downregulates the expression of LYL1 and p27kip1, the KD of *LYL1* showed induction for both BHLHE40 and p27kip1 levels and the KD of p27kip1 downregulates both LYL1 and BHLHE40 levels, three different novel feedback loops could be identified in AR signaling by SAL consisting of LYL1/BHLHE40 negative feedback loop, LYL1/p27kip1 negative feedback loop and BHLHE40/p27kip1 positive feedback loop.

Thus, LYL1 emerged as a tumor suppressor in PCa. In line with this, direct target genes of AR are negatively correlated with LYL1 expression in PCa.

## Conclusions

Androgen treatment in PCa cell lines induces BHLHE40 and LYL1 expression. Various rescue experiments indicate feedback loops in AR signaling induced by SAL, which might be relevant as biomarkers for the bipolar androgen therapy. The large overlap of LYL1 with BHLHE40 and the AR suggests a large set of genes being controlled by LYL1, BHLHE40 and the AR, which is supported by detection of protein- protein interaction among these three factors. Further, we show that p27kip1 not only inhibits CDKs but has feedback response to the LYL1 and clock gene BHLHE40 suggesting a potential link between circadian rhythm and cell cycle control. All in all, in this study we proposed a novel AR- BHLHE40 / LYL1 / p27kip1 axis in PCa that controls SAL-mediated cellular senescence signaling.

## Supplementary Information

Supplementary Material 1.

Supplementary Material 2.

## Data Availability

No datasets were generated or analysed during the current study.

## Abbreviations

AR: Androgen receptor
BAT: Bipolar androgen therapy
CSPC: Castration sensitive prostate cancer
CRPC: Castration resistant prostate cancer
Co-IP: Co-immunoprecipitation
DEGs: Differentially expressed genes
KD: Knockdown
LYL1: Lymphoblastic leukemias1
SA β-Gal: Senescence associated beta galactosidase
SAL: Supraphysiological androgen level
