# Supplementary material for: Functional circuits of LYL1 controlled by supraphysiological androgen in prostate cancer cells to regulate cell senescence
Source: Cell Commun Signal. 2024 Dec 12;22:590. doi: 10.1186/s12964-024-01970-7 (PMC11636232; doi:10.1186/s12964-024-01970-7)
Supplement: Supplementary file 2 — Supplementary Material 2. [file 12964_2024_1970_MOESM2_ESM.pdf]

Figure S1

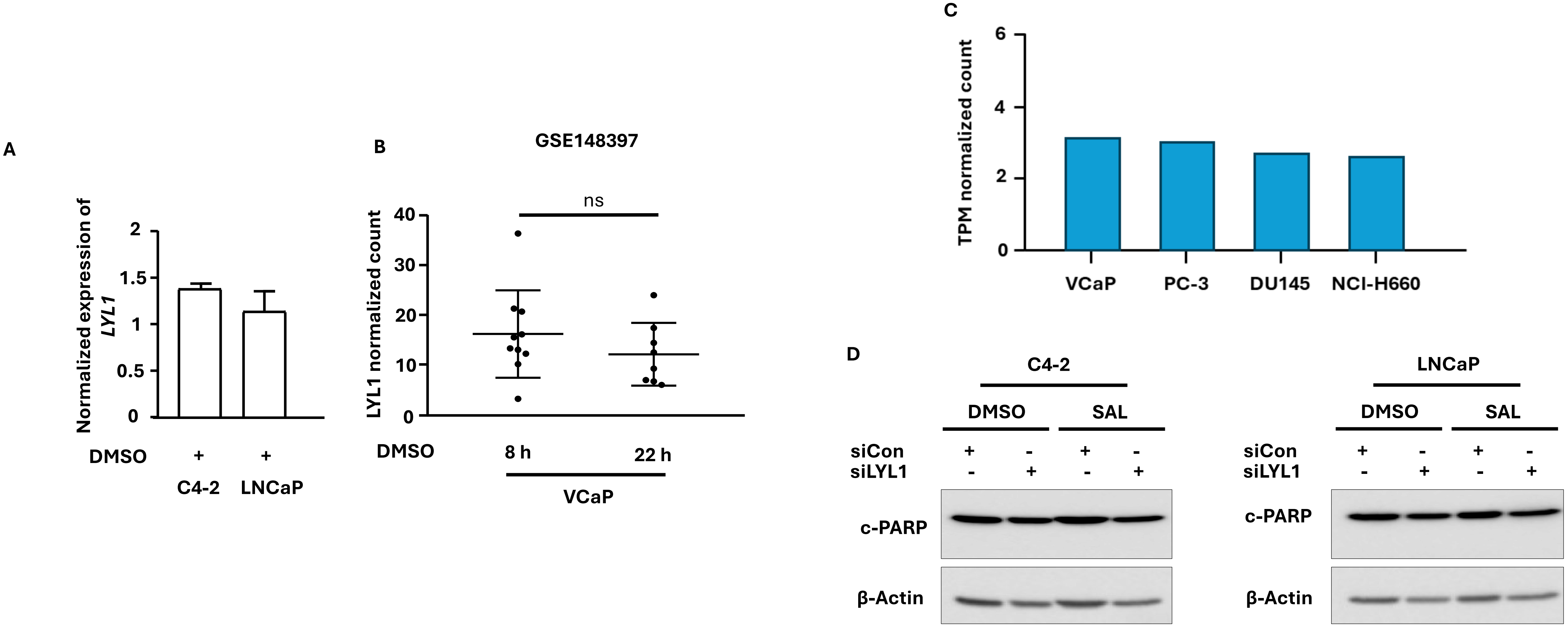

Figure S1- LYL1 mRNA levels in PCa cell lines and KD of *LYL1* does not induce apoptosis.
A: *LYL1* mRNA level in C4-2 and LNCaP cell lines with 0.1% DMSO as a solvent control. (The mRNA levels of both housekeeping genes *a-Tubulin* and *TBP* were used for normalization of expression levels). **B**: *LYL1* level in RNA-seq data from VCaP cells treated with DMSO for 8 and 22 h. Reads are DESeq2 normalized count. **C**: Expression levels of LYL1 in other PCa cell lines. Data extracted from the human protein atlas. (TPM normalized level). **D**: *LYL1* KD in both C4-2 and LNCaP cell lines does not show cleaved form of PARP as a marker of cell apoptosis.

Figure S2

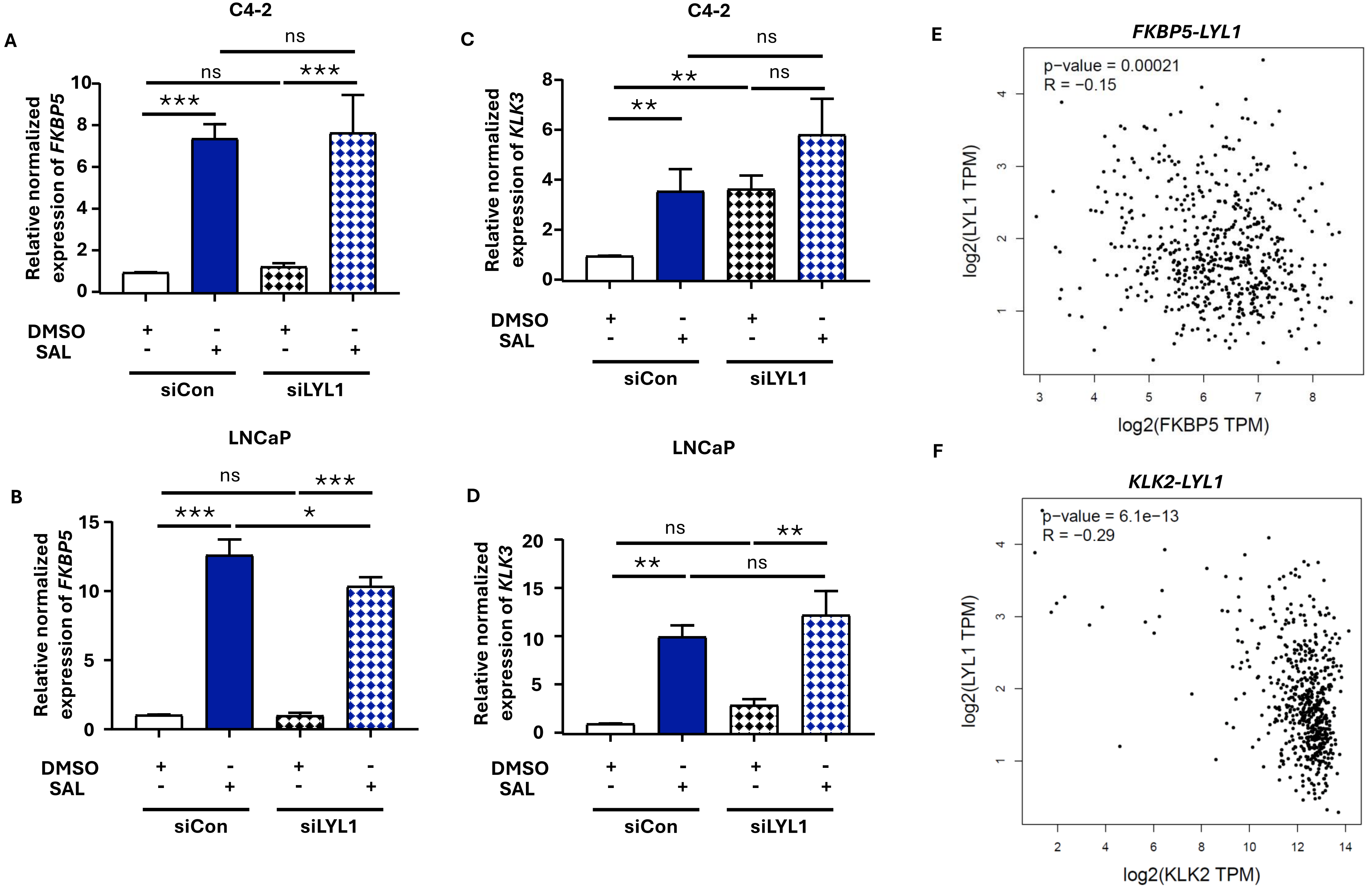
C4-2
A
Relative normalized expression of FKBP5
DMSO
SAL
siCon
siLYL1
ns
***
B
LNCaP
*
C
Relative normalized expression of KLK3
**
D
E
FKBP5-LYL1
p-value = 0.00021
R = −0.15
log2(LYL1 TPM)
log2(FKBP5 TPM)
F
KLK2-LYL1
p-value = 6.1e−13
R = −0.29
log2(KLK2 TPM)

Figure S2

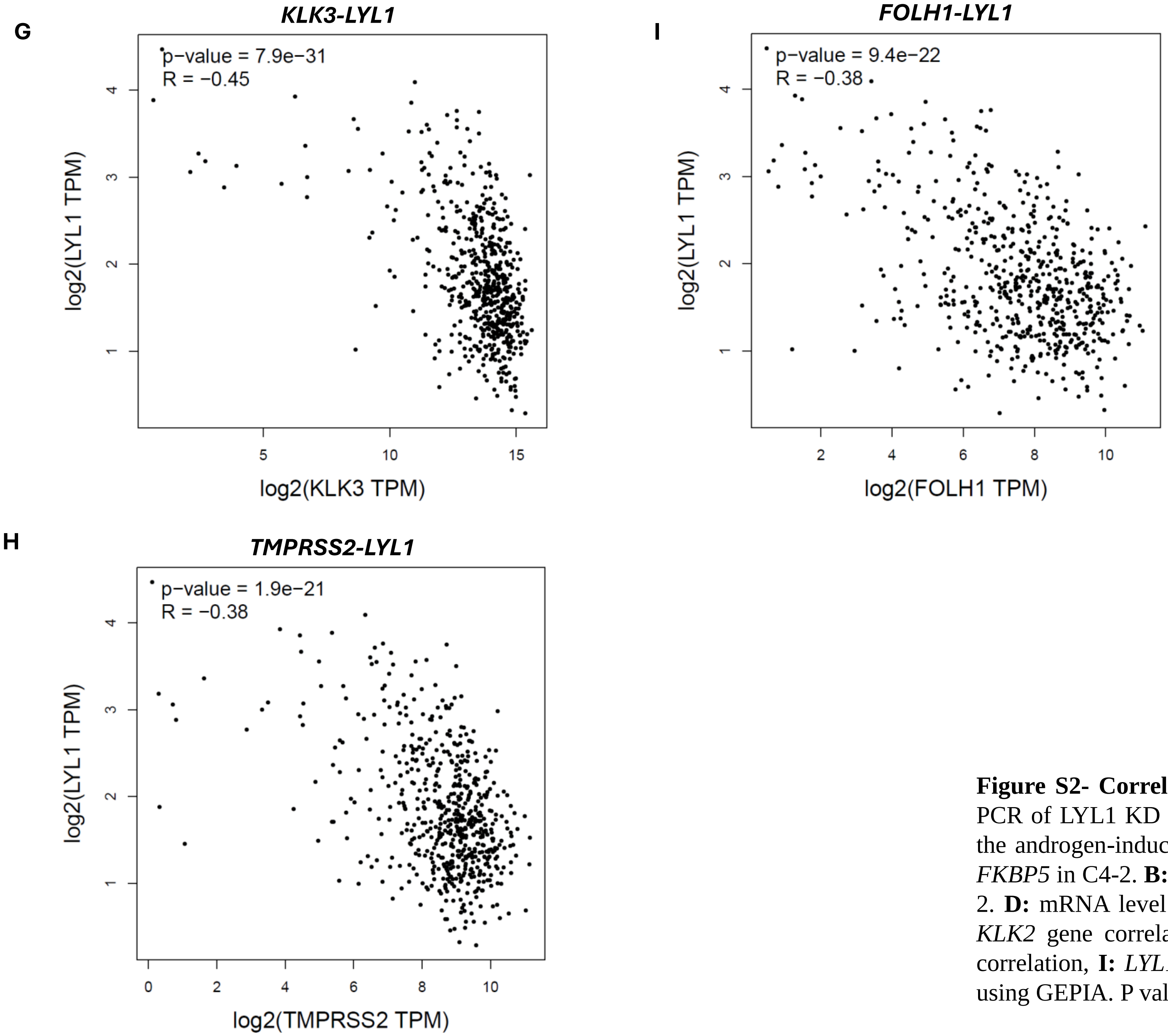

Figure S2- Correlation and regulation of AR direct target genes by LYL1. qRT-PCR of LYL1 KD cells treated with DMSO or SAL does not show strong changes in the androgen-induced expression of AR direct target genes (n=3). **A:** mRNA level of *FKBP5* in C4-2. **B:** mRNA level of *FKBP5* in LNCaP. **C:** mRNA level of *KLK3* in C4-2. **D:** mRNA level of *KLK3* in LNCaP. **E:** *LYL1-FKBP5* gene correlation, **F:** *LYL1-KLK2* gene correlation, **G:** *LYL1-KLK3* gene correlation, **H:** *LYL1-TMPRSS2* gene correlation, **I:** *LYL1-FOLH1* gene correlation. Correlation analysis was performed by using GEPIA. P value 0.001 = ***, <0.01 = **, <0.05 = *, ns= non-significant.

Figure S3

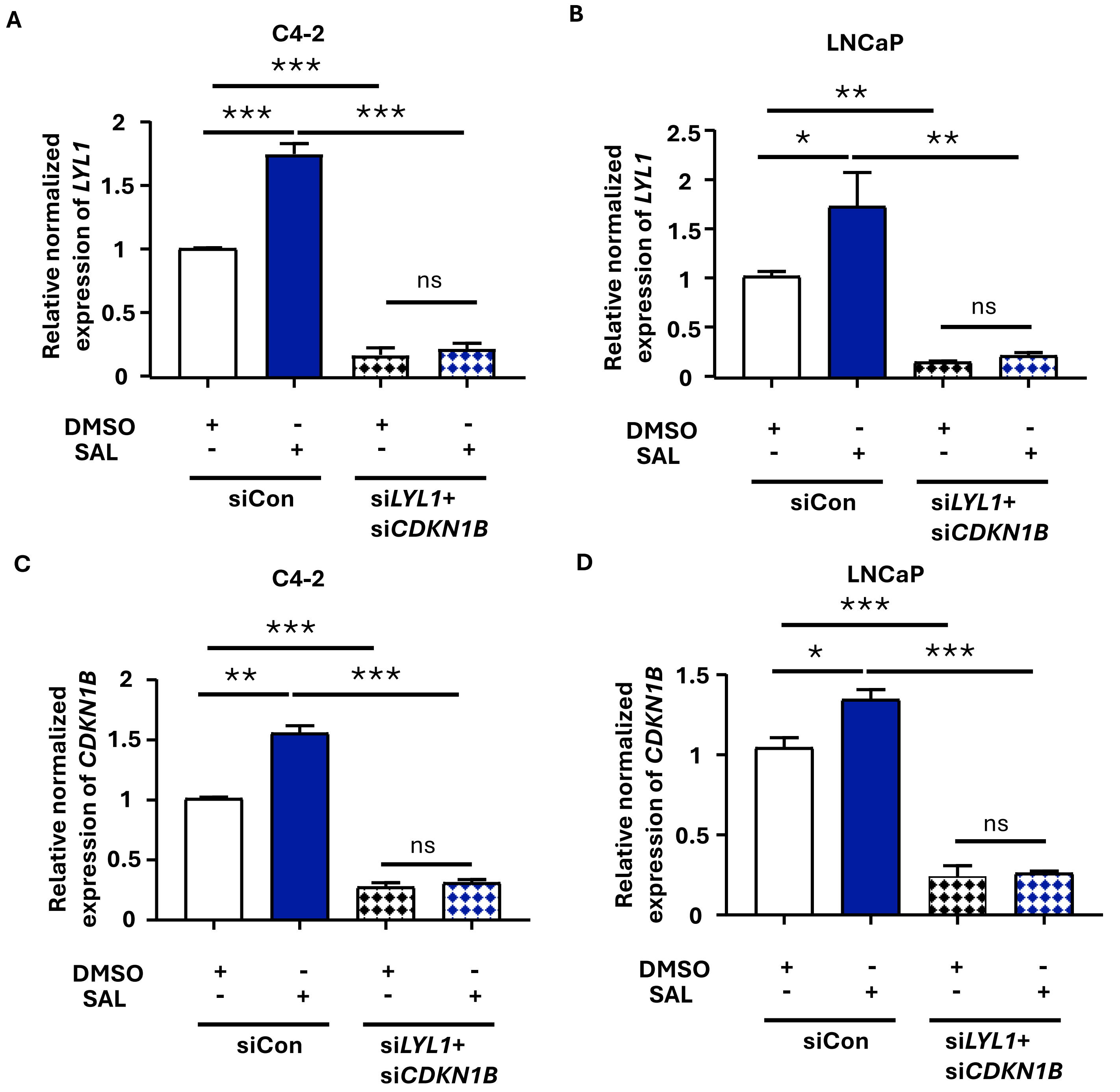

Figure S3- *LYL1* and *CDKN1B* dKD efficiency and its effects on *BHLHE40*. A and **B:** qRT-PCR of CRPC (C4-2) and CSPC (LNCaP) cells to detect *LYL1* mRNA levels with and without SAL treatment (1 nM R1881 (SAL), DMSO, respectively) and with and without dKD (si*LYL1* + si*CDKN1B* and siCon, respectively). **C** and **D:** *CDKN1B* mRNA levels in si*LYL1* + si*CDKN1B* dKD samples in C4-2 and LNCaP cell lines. P value 0.001 = ***, <0.01 = **, <0.05 = *, ns= non-significant.

Figure S4

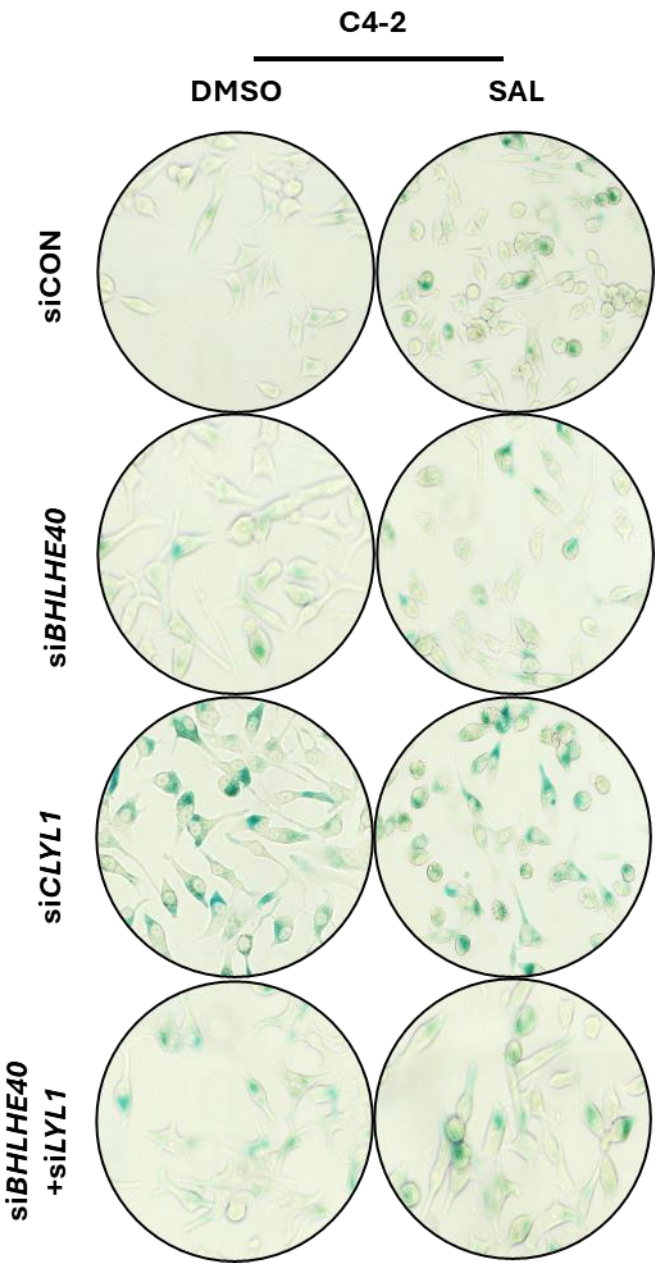

Figure S4- dKD of *BHLHE40* and *LYL1* rescued the cellular senescence induction by *LYL1* single KD. Representative SA β-Gal staining pictures for BHLHE40, LYL1 and dKD of both BHLHE40 and LYL1 in C4-2 cell line .

Figure S5

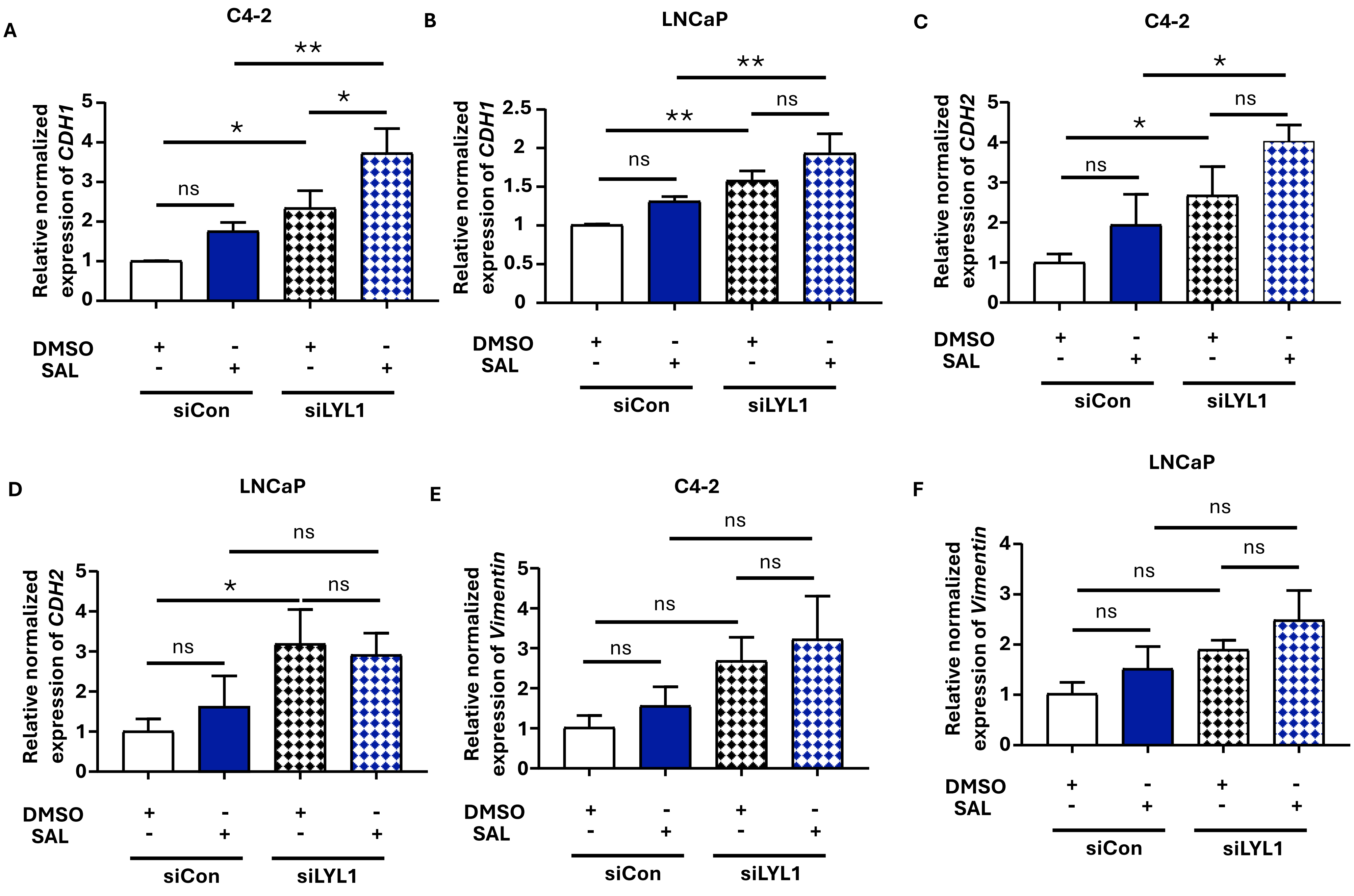

Figure S5- qRT-PCR in both cell lines represents mRNA level of EMT markers after *LYL1* KD. **A** and **B:** *CDH1* expression level in C4-2 and LNCaP. **C** and **D:** *CDH2* expression level in C4-2 and LNCaP. **E** and **F:** Vimentin expression level in C4-2 and LNCaP (n=3). P value <0.05 = *, ns= non-significant.
